# In Vitro and In Silico Antiviral Activity of Di-Halogenated Compounds Derived from L-Tyrosine against Human Immunodeficiency Virus 1 (HIV-1)

**DOI:** 10.3390/cimb45100516

**Published:** 2023-10-09

**Authors:** Maria S. Serna-Arbeláez, Valentina García-Cárcamo, Daniel S. Rincón-Tabares, Diego Guerra, Vanessa Loaiza-Cano, Marlen Martinez-Gutierrez, Jaime A. Pereañez, Manuel Pastrana-Restrepo, Elkin Galeano, Wildeman Zapata

**Affiliations:** 1 Grupo Infettare, Facultad de Medicina, Universidad Cooperativa de Colombia, Medellín 050001, Colombia; suleny.serna@udea.edu.co (M.S.S.-A.); valentina.garciac@udea.edu.co (V.G.-C.); 2 Grupo Inmunovirología, Facultad de Medicina, Universidad de Antioquia UdeA, Medellín 050001, Colombia; dsantiago.rincon@udea.edu.co; 3 Instituto de Parasitología y Biomedicina “López-Neyra”, Consejo Superior de Investigaciones Científicas, PTS Granada, 18016 Granada, Spain; diegoguerra@correo.ugr.es; 4 Programa de Estudio y Control de Enfermedades Tropicales PECET, Faculty of Medicine, University of Antioquia, Medellín 050010, Colombia; 5 Grupo de Investigación en Ciencias Animales-GRICA, Facultad de Medicina Veterinaria y Zootecnia, Universidad Cooperativa de Colombia, Bucaramanga 680005, Colombia; vanessa.loaiza@udea.edu.co (V.L.-C.); marlen.martinezg@campusucc.edu.co (M.M.-G.); 6 Grupo Toxinología, Alternativas Terapéuticas y Alimentarias, Facultad de Ciencias Farmacéuticas y Alimentarias, Universidad de Antioquia UdeA, Medellín 050001, Colombia; jaime.pereanez@udea.edu.co; 7 Productos Naturales Marinos, Departamento de Farmacia, Facultad de Ciencias Farmacéuticas y Alimentarias, Universidad de Antioquia UdeA, Medellín 050001, Colombia; mhpr2017@gmail.com (M.P.-R.); elkin.galeano@udea.edu.co (E.G.)

**Keywords:** HIV-1, antiviral, cytotoxicity, molecular docking, L-tyrosine derivatives

## Abstract

HIV-1 infection is considered one of the major public health problems worldwide. Due to the limited access to antiretroviral therapy, the associated side effects, and the resistance that the virus can generate, it has become necessary to continue the development of new antiviral agents. The study aimed to identify potential antiviral agents for HIV-1 by evaluating the in vitro and in silico activity of 16 synthetic di-halogenated compounds derived from L-Tyrosine. The compounds were tested for cytotoxicity, which was determined using MTT, and a combined antiviral screening strategy (pre- and post-infection treatment) was performed against R5 and X4 strains of HIV-1. The most promising compounds were evaluated against a pseudotyped virus (HIV-GFP-VSV-G), and the effectiveness of these compounds was measured through GFP flow cytometry. Also, the antiviral effect of these compounds was evaluated in PBMCs using flow cytometry and ELISA for p24. The TODB-2M, TODC-2M, TODC-3M, and YDC-3M compounds showed low toxicity and significant inhibitory activity against HIV-1. In silico docking and molecular dynamics assays suggest that the compounds’ antiviral activity may be due to interaction with reverse transcriptase, viral protease, or envelope gp120.

## 1. Introduction

Human immunodeficiency virus (HIV) is responsible for one of the most devastating human pandemics. According to the United Nations Program on the HIV/AIDS (UNAIDS) report, in 2022, 39 million people were living with HIV, and 1.3 million contracted the infection last year. An estimated 29.8 million people (76.4% of those infected) had access to antiretroviral therapy. Despite global efforts to reduce HIV/AIDS-related deaths and the implementation of antiretroviral therapy (ART), there were nearly 630,000 deaths from acquired immunodeficiency syndrome (AIDS)-related illnesses by 2021 [1].

Combination antiretroviral therapy (cART) successfully suppresses HIV viral load, increases the number of CD4+ T lymphocytes, restores immune function, prevents HIV transmission, avoids resistance, prevents clinical progression of the disease, and improves the quality of life [2]. cART acts on different steps of the HIV replication cycle. This therapy includes two nucleoside reverse transcriptase inhibitors (NRTIs) combined with an integrase inhibitor, a protease inhibitor (PI), or a non-nucleoside reverse transcriptase inhibitor (NNRTI) [3,4].

The lack of adherence can affect the total inhibition of viral replication, favoring the emergence of resistant strains [5]. This resistance to cART can be primary or transmitted at the time of infection, in the case of patients who have not been previously exposed to the drug, or secondary or acquired resistance, which has been described in patients taking these drugs [5,6]. For notwithstanding treatment with effective drugs and even when treatment adherence is maintained, some resistance to anti-HIV cART is expected to emerge. This drug resistance differs depending on the class of antiretroviral drugs, as some have a higher genetic barrier than others [6,7]. HIV drug resistance can affect the efficacy of cART and the duration of the drugs available to treat HIV infection.

Thus, the continued development of new drugs with high potency against new therapeutic targets, with improved efficacy and safety profile, is a priority. Natural products have attracted special interest due to the existing biodiversity worldwide. Natural products and their derivatives represent an excellent option due to their therapeutic potential against HIV-1 infection. In fact, a high percentage of FDA-approved drugs are derived directly or indirectly from natural products [8]. HIV treatment has limitations; therefore, finding new anti-HIV compounds with accepted toxicity and a lower resistance profile is a pressing need.

Natural products with potential anti-HIV activity have been isolated mainly or derived from plants and marine sponges. These include terpenes, coumarins, flavonoids, laccases, lectins, ribosome-inactivating proteins (RIPs), and bromotyrosines [9].

Bromotyrosine is a chemical compound that contains a bromine atom attached to a tyrosine molecule, commonly found in marine organisms, particularly sponges. Research has shown that bromotyrosines derivatives may have various pharmacological activities, including anti-inflammatory [10], antifungal [11,12], and antitumor properties [13] and are therefore being studied as potential therapeutic agents.

Recently, Galeano et al. reported the isolation of bromotyrosine-derived compounds from the Colombian Caribbean marine sponge species *Aiolochroia crassa* and *Verongula rigida*, which showed pharmacological potential as inhibitors of HIV-1 replication in vitro [14]. Likewise, it has been reported that some di-halogenated phenolic compounds derived from L-tyrosine show activity against Zika and Chikungunya viruses.

In this context, the active search for compounds that can be used for the treatment of HIV is a worldwide research priority. This work evaluated the in vitro and in silico anti-HIV-1 activity of sixteen di-halogenated compounds derived from L-Tyrosine.

## 2. Materials and Methods

### 2.1. Di-Halogenated Compounds Derived from L-Tyrosine

Sixteen di-halogenated compounds derived from L-tyrosine were evaluated. The di-halogenation compounds were performed with substitution on the phenyl ring at positions 3 and 5, as previously described [15]. All compounds were dissolved in a water solution with 5% dimethyl sulfoxide (DMSO) (Sigma-Aldrich, St Louis, MO, USA) at a 1 mg/mL concentration. All the compounds synthesized were analyzed via nuclear magnetic resonance and high-resolution mass spectrometry. All the NMR spectra showed purity higher than 98% (Supplementary Figures S1–S16). The compounds were classified into two groups: L-Tyrosine derivates and Tyramine derivates (Figure 1).

### 2.2. Cells Lines and Culture Conditions

TZM-bl cells were obtained from the NIH AIDS Research and Reference Reagent Program (Cat. no. 8129). This cell line is derived from a clone of HeLa cells designed for expressing HIV-1 virus entry receptor and co-receptors (CD4, CXCR4, and CCR5) with an integrated luciferase gene under the control of the HIV-1 promoter long terminal repeat (LTR) [16,17], which makes it possible to measure infection. HEK293T cells were purchased from the American Tissue Culture Collection (ATCC). TZM-bl and HEK293T cells were cultured at 37 °C with 5% CO_2_ in DMEM supplemented with heat-inactivated fetal bovine serum (FBS) (Sigma-Aldrich, St. Louis, MO, USA). Cell monolayers were detached via treatment with a Trypsin ethylenediaminetetraacetic acid (EDTA) solution of 0.25% (Sigma-Aldrich, St. Louis, MO, USA).

H9/HTLV-IIIB cells (ATCC-CRL-8543) were persistently infected and were used to produce HIV-1IIIB (X4-tropic HIV-1) viral stocks. Human peripheral blood mononuclear cells (PBMC) from two healthy donors were obtained from the blood bank of the Hospital Universitario San Vicente Fundación. PBMC were isolated through a density gradient with Ficoll-Histopaque (Sigma-Aldrich, St. Louis, MO, USA). H9/HTLV-IIIB and PBMC were maintained in RPMI-1640 medium (Sigma-Aldrich, St. Louis, MO, USA), supplemented with 10% heat-inactivated FBS incubated in a humid atmosphere with 5% CO_2_ at 37 °C.

### 2.3. Virus Strains

R5-tropic HIV-1 was obtained through the NIH HIV Reagent Program, Division of AIDS, NIAID, NIH: Human Immunodeficiency Virus Type 1 (HIV-1) BaL, ARP-510, contributed by Dr. Suzanne Gartner, Dr. Mikulas Popovic, and Dr. Robert Gallola [18]. X4-tropic HIV-1IIIB was obtained from the HIV-1 chronically infected human T cell line IIIB (H9/HTLV-IIIB) (ATCC-CRL-8543). Pseudotyped HIV-1 virus stocks (HIV-GFP-VSV-G reporter virus) were produced in HEK293T cells using lipofectamine 3000 (Invitrogen, Waltham, MA, USA) co-transfection of pNL4-3 delta env GFP (HIVΔenv.GFP) and pVSV-G plasmids encoding for full-length HIV-1 NL4-3 proviral DNA, with a frameshift in the env gene and expressing GFP instead of nef and for vesicular stomatitis virus G protein, respectively. These plasmids were kindly provided by Johnny He [19,20].

### 2.4. Cytotoxicity Assay

The in vitro cytotoxic effect of the di-halogenated compounds on TZM-bl cells was determined with the MTT (3-(4,5-Dimethylthiazol-2-yl)-2,5-diphenyltetrazolium bromide) assay (Sigma-Aldrich, St. Louis, MO, USA). For this purpose, 1.2 × 10^5^ cells/well were seeded in 96-well plates, which were incubated at 37 °C and 5% CO_2_ for 24 h. DMEM was used as a negative control and serial two-fold dilutions of the compounds were performed at 9.37 to 300 μM in DMEM supplemented with 5% FBS. A total of 100 μL/well of each dilution was added and incubated for 48 h. Subsequently, the cells were washed 2 times with 1X PBS, and 30 μL of MTT solution (2 mg/mL) was added and incubated for 2 h at 37 °C, in a light-protected culture. Then, 100 µL of DMSO was added to dissolve the formazan crystals, placed in shaking for 15 min, and absorbance reading was performed at 570 nm, using the Microplate Spectrophotometer (Thermo Scientific™ Multiskan™ GO, Waltham, MA, USA). Two independent experiments evaluated each experimental condition in quadruplicate (*n* = 8). The percentage of viability was calculated concerning the absorbance of the controls without a compound (100% viability). The 50% cytotoxic concentration was defined as the concentration of each compound that can reduce cell viability to 50% of the untreated cell control. CC_50_ was calculated and analyzed with GraphPad Prism version 8.0.1 (GraphPad Software, Inc., San Diego, CA, USA).

### 2.5. Viability Assay

PBMCs were thawed and let in the culture in RPMI with 10% fetal bovine serum (FBS) (Gibco, Grand Island, NY, USA) for 24 h before each experiment. Cells were stimulated for 48 h with 8 μg/mL of Phytohemagglutinin-PHA (Sigma-Aldrich, St. Louis, MO, USA) and 50 UI/mL of IL-2 (Peprotech, Rocky Hill, CT, USA). In the presence of various concentrations of di-halogenated compounds derived from L-Tyrosine, 2 × 10^5^ cells per well were treated and resuspended in 200 µL of fresh medium with 20 UI/mL of IL-2 and incubated in 96-well culture plates for 7 days. After 7 days post-treatment, cell viability was determined using flow cytometry. Briefly, cells were stained with fixable viability dye eFluor 506 (eBioscience, Leicestershire, UK) as detailed in the manufacturer’s protocol for 30 min in the dark. Later, cells were washed and resuspended in PBS, and the percentage of viable cells was measured instead in the LS Fortessa cytometer (BD Biosciences, Franklin Lakes, NJ, USA). Data were analyzed using FlowJo version 10.5.3 (FlowJo, LLC, Ashland, OR, USA).

### 2.6. In Silico Toxicological Modeling/ADMET Profiles

In silico toxicological modeling of halogenated molecules derived from L-Tyrosine was performed using ADMET Predictor^®^ v8 software from Simulation Plus [21], which predicts toxicity parameters in different biological models. For this, the structures of the molecules were modeled in the ACD/ChemSketch ^®^12.01 (Freeware Version) program, the geometry was optimized using the Avogadro software, and they were subsequently entered into the ADMET Predictor^®^ software, which issued the results of the parameters of interest defined in the study. For the toxicological evaluation, in addition to the compounds and control drugs evaluated, we used a compound that was called “ideal”, which is hypothetical, to compare the toxicity among all the compounds. With these results, a hierarchical cluster analysis (Ward’s method and Bray–Curtis similarity index) was performed with R [22]. Moreover, the score of the potential toxicity level of each compound was defined on a scale from 0 to 13 and was reported as cumulative toxicity, according to the sum of the qualitative result of each parameter, whether it was toxic (T)—one (1) point—or non-toxic (N)—zero (0) points—and was classified as low: 1 to 4 positive toxic parameters, medium: 5 to 8 positive toxic parameters, or high: 9 to 13 positive toxic parameters.

### 2.7. Anti-HIV Activity in Infected TZM-bl Cells

TZM-bl cells were plated in a flat-bottom 96-well plate (Corning Inc., Corning, New York, NY, USA) at the concentration of 1.2 × 10^5^ cells/well and incubated for 24 h at 37 °C before the addition or not of each compound at non-cytotoxic concentrations (pre-infection treatment). Then, 1 h later the treatment was removed, and the infection was performed by adding 10 ng/mL of HIV-1IIIB p24 (X4) or 2 ng/mL of HIV-1 BaL (R5). After 3 h, the virus was removed and washed, and the compound dilutions were added again (post-infection treatment). Additionally, three wells were left for the negative control (cells without treatment or infection), the positive control of infection (cells infected with the virus without treatment), and the positive control of inhibition (AZT). Antiviral activity was quantified 48 h after treatment by measuring luciferase activity, which is directly proportional to the number of infectious virus particles in the sample [23]. The luciferase activity was quantified via luminescence emitted by the cells using Bright-GloTM Luciferase Assay System (Promega Corp., Madison, WI, USA), following the manufacturer’s instructions. Briefly, 100 μL of the reagent was added to 100 μL of TZM-bl cells and incubated for at least 2 min, protected from light. Relative light units (RLUs) were measured using the multi-mode plate reader (Thermo ScientificTM VarioskanTM LUX). Each experimental condition was performed in triplicate in two independent experiments (*n* = 6). The percentage inhibition was calculated as follows: 100 − (RLU of treated cells − RLU of uninfected cells/RLU of infection control − RLU of uninfected cells) × 100. The IC_50_ was defined as the compound concentration that can reduce viral infection to 50% of the infected control cells not treated with the compounds.

### 2.8. VSV-Pseudotyped HIV-1 Infection Assay and Quantification of the Percentage of Infected Cells Using Flow Cytometry

TZM-bl cells were plated in a 96-well plate at the concentration of 1.2 × 10^5^ cells/well 1 day before infection, then inoculated with HIV-GFP-VSV-G reporter virus stocks (40 ng/mL of p24) in the presence of 10 µg/mL polybrene (Sigma-Aldrich, St Louis, MO, USA) for 3 h. Cells were washed twice and treated with the most bioactive compounds at non-cytotoxic concentrations for 48 h; finally, cells were trypsinized and rewashed with PBS. Antiviral activity was quantified using flow cytometry for GFP expression. Cells were acquired using LS Fortessa (BD Biosciences, San Jose, CA, USA), and data were analyzed using FlowJo version 10.5.3 (FlowJo, LLC, Ashland, OR, USA). The percentage of inhibition was calculated as follows: 100 − (Infection percentage in treated cells × 100/percentage in control infection).

### 2.9. Anti-HIV Activity of Di-Halogenated Compounds Derived from L-Tyrosine on HIV Infection of PBMC

In the presence of various concentrations of the most bioactive compounds, 1×10^6^ PBMCs previously stimulated with 8 μg/mL of PHA and 50UI/mL of IL-2 were treated for 1 h. After, cells were infected via spinoculation for 90 min at 700× *g* with 1ng/mL of HIV-1BaL p24/million cells. After spinoculation, cells were washed twice with PBS 1x to remove free virions, and then, fresh medium supplemented with 20 UI/mL of IL-2, with or without the di-halogenated compounds derived from L-Tyrosine dilutions, was added. Cells were plated in 96-well culture plates and incubated for 7 days. After 7 days, viral production was assessed in the supernatant using HIV-1 p24 ELISA (Xpressbio, Frederick, MD, USA). In addition, antiviral activity was also assessed by measuring intracellular p24 in harvested cells. Briefly, cells were stained with fixable viability dye eFluor 506 (eBioscience, Leicestershire, UK), CD3 (UCHT1; Thermo Scientific, Wilmington, DE, USA), CD4 (RPA-T4; BD Biosciences, San Jose, CA, USA), and p24 PE (KC57; Beckman Coulter, Pasadena, CA, USA), as detailed in the manufacturer’s protocol. Later, cells were washed and resuspended in PBS, and the lecture was measured instead in the LS Fortessa cytometer (BD Biosciences, Franklin Lakes, NJ, USA). Data were analyzed using FlowJo version 10.5.3 (FlowJo, LLC, Ashland, OR, USA).

### 2.10. Quantification of HIV-1 p24 Antigen Using ELISA

The supernatants obtained in the production of the different viral stocks and those obtained in the assay of anti-HIV activity in PMBCs were collected for titration via p24 estimation using an ELISA kit (XpressBio, Frederick, MD, USA), following the manufacturer’s instructions.

### 2.11. Molecular Docking Simulation

The structure of the di-halogenated compounds derived from L-tyrosine was obtained with the program ACD/ChemSketch ^®^12.01 (Freeware Version), and the structure of the control compounds was from the DrugBank database https://www.drugbank.ca/ (accessed on 2 August 2022). The geometry of all compounds was optimized using Avogadro software [24,25]. The ligands preparation for docking was performed using the Autodock tools (ADT) program [26].

For molecular docking, three-dimensional structures of seven structural and non-structural viral proteins were downloaded from the PDB (Protein Data Bank), and structures with a resolution equal to or less than 2.5 Å were considered. The seven HIV-1 viral proteins chosen for analysis were gp120 (PDB: 4J6R), gp41 (PDB: 3VTQ), RT (PDB: 4G1Q), IN (PDB: 1QS4-3OYA), protease (PDB: 5YOK), p17 (PDB: 4JMU), and p24 (PDB: 2XDE). For the integrase structure, the 1QS4 structure, which has one of the Mg^2+^ of the active site, was initially taken as a basis. The missing residues were incorporated [27], and the second magnesium ion was placed in the same relative position according to the two metal structures of the Prototype Foamy Virus integrase (PDB code 3OYA) [28]. The 3D models of interest were prepared for docking using the Python Molecular Viewer (PMV) software [29]. To determine the possible molecule binding sites on these proteins, the PeptiMap server [30,31] was used, and based on these sites, box coordinates were defined and evaluated with the exhaustiveness of 10. Finally, to determine the best interactions between the viral proteins and the compounds, the Autodock Vina software [32] was used. Evaluation of possible interactions was performed using PMV and Discovery Studio software [33]. Table 1 shows the viral proteins evaluated in the study and the amino acids that were used as targets for molecular docking.

### 2.12. Molecular Dynamics Simulations

The structures of RT and Protease bound to the TODC-2M and TODB-M compounds from the molecular docking analyses were subjected to molecular dynamics (MD) simulations of 100 ns. A dodecahedron box of water surrounded the protein with a temperature of 310 K. Before the MD, the structures were minimized and subjected to NVT/NPT equilibration phases using GROMACS v2022.1 [34]. During the MD phases, the CHARMM36 force field [35], a modified Berendsen thermostat [36], and the Parrinello–Rahman barostat [37] were used. The complex was solvated with periodic boundary conditions at a distance of at least 10 Å, and counterions were included in the solvent to make the box neutral. The Particle Mesh Ewald (PME) method was used to calculate the electrostatic interactions with 1.0 nm short-range threshold. We used for the simulations a timestep of 2 femtoseconds (fs). Because the ligands are not part of the selected CHARMM36 force field, the server CGenFF was used to parameterize the molecules for the MD analysis [35].

After running the simulations, 2000 frames per trajectory were extracted to perform the AutoDock Vina scoring [32] for each frame, calculating a cumulative average score. This assessment helped evaluate the potential affinity of the ligands, indirectly considering the protein flexibility.

### 2.13. Statistical Analysis

The results of the experiments will be expressed as means ± SD of at least 2 independently performed experiments. Differences between groups were analyzed using one-way analysis of variance (ANOVA) since the data were normally distributed, and statistically significant differences with a *p*-value less than 0.05 were considered for all cases. Data were analyzed using the GraphPad Prism v.8.0.1 software (San Diego, CA, USA).

## 3. Results

### 3.1. Di-Halogenated Compounds Derived from L-Tyrosine Have No Significant Cytotoxic Effect on TZM-bl Cells

**Figure 1:**
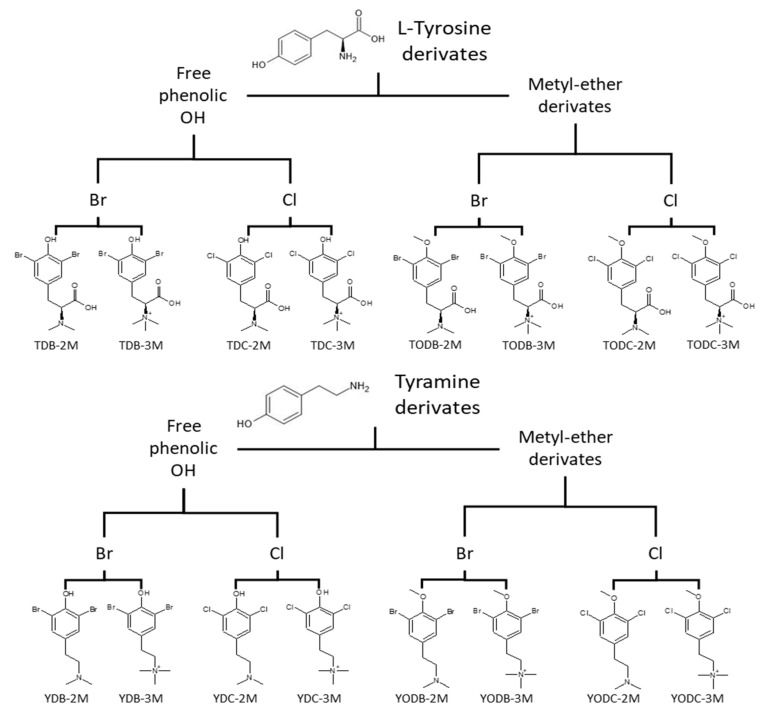
Classification of evaluated compounds. Sixteen di-halogenated compounds derived from L-tyrosine were evaluated, which were classified into derived from L-tyrosine and derived from tyramine. These groups were further subdivided into compounds with free phenolic OH and methyl ether derivatives. Finally, each subgroup was disrupted into compounds classified by halogen type: bromine (Br) or chlorine (Cl).

A total of sixteen phenolic di-halogenated compounds, synthesized from L-tyrosine as shown in Figure 1, were subjected to in vitro toxicity assessments to determine their optimal concentrations for use in antiviral experiments.

**Figure 2:**
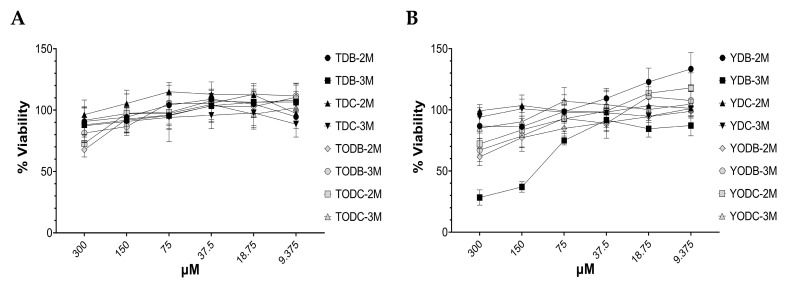
Percentage viability of TZM-bl cells treated with tyrosine-derived di-halogenated compounds. (**A**). L-tyrosine. (**B**). Tyramine derivatives. TZM-bl cells were treated with different concentrations of each compound (9.37 μM up to 300 μM for 48 h). Percentage viability was calculated relative to untreated cells. Each sample was tested in triplicate in two independent experiments; the results are shown as mean ± standard deviation.

At a concentration of 150 μM, all the tyrosine-derived compounds displayed a cytotoxicity level of ≤10% (with a viability of greater than or equal to 90%), except for TODB-3M, which had a lower cytotoxicity level of less than 20% (Figure 2A). Conversely, all the tyramine-derived compounds demonstrated a cytotoxicity level of ≤30% (with a viability of greater than or equal to 77%) at the same concentration, except for YDB-3M, which exhibited a higher level of cytotoxicity (Figure 2B). Based on the low cytotoxicity observed for the tested compounds and their availability, subsequent assays were conducted using non-cytotoxic concentrations. Neither the viral inhibition control (AZT) nor the solvent (DMSO) was toxic.

### 3.2. In Silico Prediction Indicates That Di-Halogenated Compounds Are Not Highly Toxic

In silico toxicity modeling for all compounds and control drugs was performed with ADMET Predictor^®^, and the findings are described in Table 2.

None of the compounds or controls exhibited high toxicity in terms of accumulated toxicity. L-tyrosine derivatives had low toxicity, except for TODB-2M, while tyramines had medium toxicity, except for YODC-2M and YODC-3M, which had a low toxicity. The compound that was predicted to be the most toxic based on toxicity parameters was YDB-2M with a score of 7, like the control drugs efavirenz and zidovudine, which had scores of 8.

Table 2 presents data on global risks (ADMET-Risk) calculated using the Lipinski rules. All compounds had a minimal global risk score of less than 7, except for the control drugs lopinavir and ritonavir, which scored 7.5 and 9.7, respectively. Lipinski’s rule-of-five analysis was also performed on the Molinspiration Cheminformatics server in this study. All compounds evaluated in the study were satisfied with Lipinski’s rule of five, as they all had a molecular mass below 500 Daltons, less than 5 hydrogen bond donors, less than 10 hydrogen bond acceptors, and had high lipophilicity (expressed as LogP below 5). The molar refractivity was between 40 and 130 (Supplementary Table S1). Overall, the compounds and controls evaluated in this study had relatively low toxicity and risk levels.

**Figure 3:**
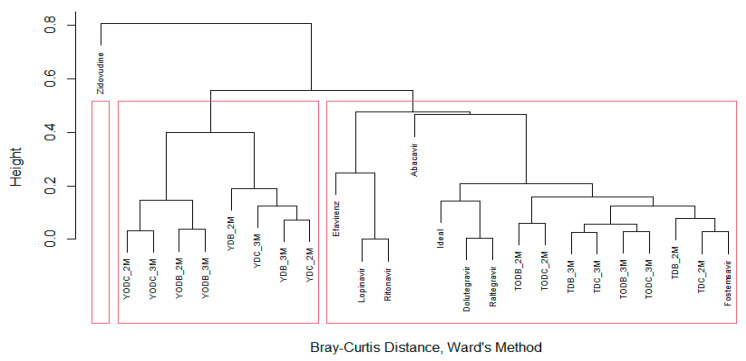
Cluster analysis of di-halogenated compounds. Hierarchical cluster analysis (Ward’s method and Bray–Curtis similarity index) based on the toxicity parameters of the different compounds, evaluated using ADMET predictor^®^.

In addition to the parameters outlined in Table 1, a hierarchical cluster analysis (using Ward’s method and Bray–Curtis similarity index) was conducted with R to assess the biological activity of the ADMET predictor. The analysis revealed the formation of three clusters (shown in Figure 3). The most promising compounds in terms of low toxicity were found in the same group as the “ideal” compound, which included L-tyrosine derivatives and the control drugs dolutegravir, raltegravir, and fostemsavir. These compounds had the lowest cumulative toxicity. The second cluster contained all tyramine derivatives, which were generally more toxic than L-tyrosine derivatives, with cumulative toxicity ranging from four to seven points. Finally, zidovudine was placed in the third cluster and exhibited significant differences from the other compounds in terms of toxicity level.

### 3.3. Some Di-Halogenated Compounds Inhibit Replication of HIV-1 BaL (R5 Strain)

**Figure 4:**
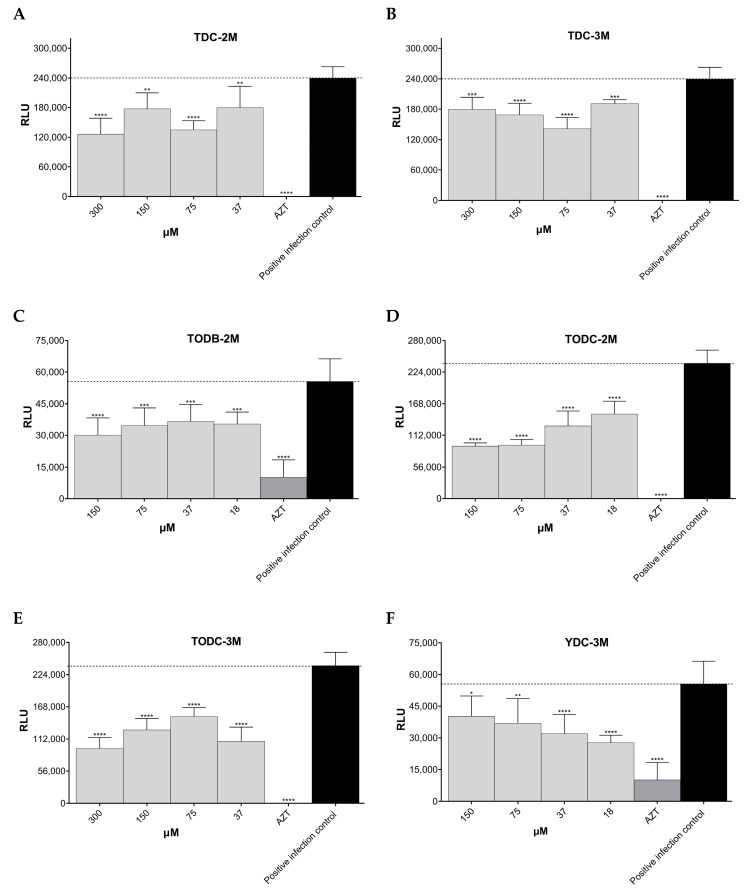
Antiviral activity of di-halogenated compounds on HIV-1 BaL replication. (**A**). TDC-2M. (**B**). TDC-3M. (**C**). TODB-2M. (**D**). TODC-2M. (**E**). TODC-3M. (**F**). YDC-3M. AZT (10 µM) was used as a control of replication inhibition. Each sample was tested in triplicate in two independent experiments; the results are shown as mean ± standard deviation. The signs *, **, ***, and **** above the columns mean *p* < 0.05, *p* < 0.01, *p* < 0.001, and *p* < 0.0001, respectively, and correspond to the comparison with the positive infection control. The dotted line indicates the RLU of the positive infection control.

The percentage of HIV-1 BaL replication inhibition for each compound at different concentrations obtained via luminescence is shown in Supplementary Table S2. The di-chlorinated compounds TDC-2M (IC_50_ 140.7 µM) and TDC-3M showed significant inhibition of HIV-1 BaL infection, with a percentage inhibition of 47.3% at 300 µM and 39.7% at 75 µM, respectively, compared to the positive infection control, as shown in Figure 4A,B. TODB-2M (IC_50_ 113.5 µM), TODC-2M (IC_50_ 67.42 µM), and TODC-3M also showed statistically significant anti-HIV activity with a percentage inhibition of 45.8%, 61.1%, and 59.9%, respectively, at the highest concentrations tested (Figure 4C–E).

Conversely, the tyramine-derived brominated compounds YDB-2M and YDB-3M did not show significant anti-HIV activity but increased viral replication at high concentrations (Supplementary Table S2). Finally, YDC-3M (IC_50_ 37.2 µM) showed 50.2% inhibition of HIV-1 BaL infection at lower concentrations, which was statistically significant, but this effect decreased at higher concentrations (Figure 4F). The other compounds did not show statistically significant anti-HIV activity. The positive inhibition control AZT showed over 90% inhibition of viral infection at a concentration of 10 µM.

Compounds TDC-2M, TODB-2M, and YDC-3M, which were active against the R5 strain, showed an SI of ~1.24, 1.45, and 1.07, respectively (Table 3).

### 3.4. Some Di-Halogenated Compounds Inhibit Replication of HIV-1_IIIB_ (X4 Strain)

Supplementary Table S3 shows the percentage of inhibition of HIV-1 BaL replication achieved with each compound at different concentrations, measured via luminescence.

**Figure 5:**
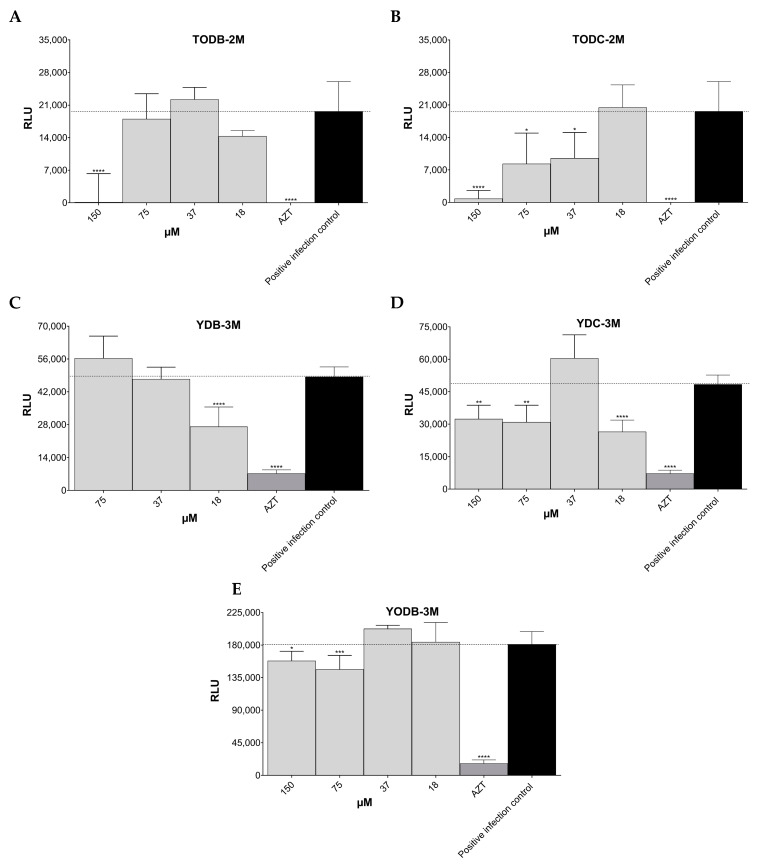
Antiviral activity of di-halogenated methyl ether L-tyrosine-derived compounds on HIV-1IIIB (X4 strain) replication. Antiviral activity was determined by measuring luciferase activity using a luminometer. (**A**). TODB-2M. (**B**). TODC-2M. (**C**). YDB-3M. (**D**). YDC-3M. (**E**). YODB-3M. AZT (10 µM) was used as a control. Each sample was tested in triplicate in two independent experiments; the results are shown as mean ± standard deviation. The signs *, **, *** and **** above the columns mean *p* < 0.05, *p* < 0.01, *p* < 0.001, and *p* < 0.0001, respectively, and correspond to the comparison with the positive infection control. The dotted line indicates the RLU of the positive infection control.

The brominated tertiary amines TODB-2M (IC_50_ ~ 82.68 µM) and chlorinated compound TODC-2M (IC_50_ ~ 39.15 µM) showed potent antiviral activity with inhibition percentages of 95.9% and 99.6%, respectively, with statistically significant differences compared to the positive infection control, when evaluated at a concentration of 150 µM (Figure 5A,B). In contrast, their respective quaternary ammonium salts did not show statistically significant inhibitory activity.

Regarding tyramine-derived compounds, the quaternary ammonium salts YDB-3M (IC_50_ 3.09 µM) and YDC-3M (IC_50_ 22.31 µM) at a concentration of 18 µM inhibited HIV-1 replication by 39.2% and 40.9%, respectively, with statistically significant differences compared to the positive infection control (Figure 5C,D). Only one tyramine-derived compound with free phenolic OH, YODB-3M, showed statistically significant antiviral activity with a percentage of inhibition of 20.3% (75 µM) (Figure 5E). The positive control AZT at a concentration of 10 µM inhibited more than 90% of virus infections in TZM-bl cells.

Compounds TODB-2M, YDB-3M, and YDC-3M, which were active against the X4 strain, showed an SI of ~1.99, ~32.65, and 1.65, respectively (Table 4).

According to the results obtained in the antiviral screening of the 16 compounds, 3 compounds (TODB-2M, TODC-2M, and YDC-3M) with the highest antiviral activity against both R5 and X4 viral strains were selected for further evaluation of their anti-HIV activity in a different viral model. Since viruses with CCR5 tropism (R5 strain) are responsible for the initial or acute HIV-1 infection, the TODC-3M compound was also chosen for this assay because it demonstrated a significant level of inhibition against the R5 strain.

### 3.5. TODB-2M and Other Di-Halogenated Compounds Inhibit Replication of HIV-GFP-VSV-G

**Figure 6:**
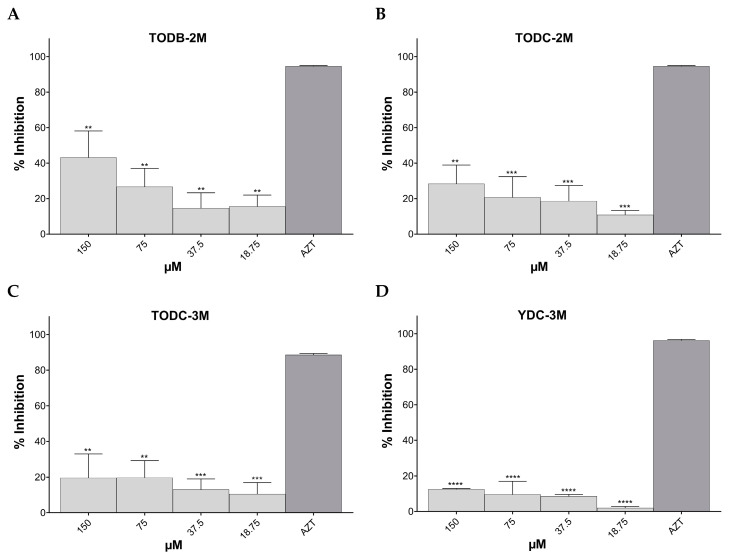
Antiviral activity of di-halogenated compounds on HIV-GFP-VSV-G in vitro replication. Antiviral activity was determined by measuring the GFP expression levels using flow cytometry. (**A**). TODB-2M. (**B**). TODC-2M. (**C**). TODC-3M. (**D**). YDC-3M. AZT (10 µM) was used as a control of viral inhibition replication. The graphs represent the median of two independent experiments performed in triplicate but measured as a single pool; the results are shown as mean ± standard deviation. The signs **, ***, and **** above the columns mean *p* < 0.01, *p* < 0.001, and *p* < 0.0001, respectively, and correspond to the comparison with the positive inhibition control (AZT).

The percentage inhibition of HIV-GFP-VSV-G replication based on data obtained by assessing the percentage of GFP+ cells using flow cytometry is shown in Figure 6.

The tested compounds TODB-2M (IC_50_ 141.8 µM), TODC-2M, and YDC-3M showed a percentage inhibition of 44.2%, 29%, and 12.5%, respectively, at the highest concentration tested (150 µM). These differences were statistically significant for all compounds compared to the infection control. All three compounds inhibited HIV-1 replication in a dose-dependent manner. Compound TODC-3M inhibited infection by 19.5%, but it was not significantly different from the infection control. The positive inhibitory control, AZT, inhibited 93.2% of viral replication (Figure 6).

Compound TODB-2M, which was active against a pseudotyped, virus obtained a selectivity index of 1.16 (Table 5).

### 3.6. The Most Promising Compounds Have no Impact on the Cell Viability of PBMC

**Figure 7:**
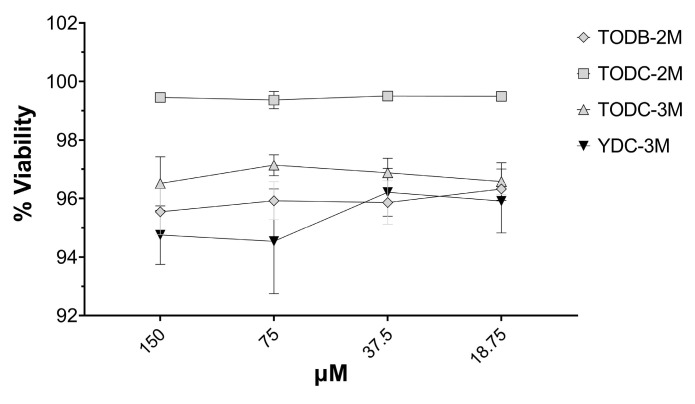
PBMCs were treated with the most promising compounds. PBMCs were treated with different concentrations of each compound (18.75 μM up to 150 μM for 7 days). Percentage viability was calculated relative to untreated cells. Each sample was tested in quadruplicate in two independent experiments; the results are shown as mean ± standard deviation.

The tyrosine-derived compounds TODB-2M, TODC-2M, TODC-3M, and tyramine YDC-3M demonstrated >90% viability level on PBMCs at all concentrations tested (Figure 7). Considering the low cytotoxicity observed, subsequent assays were performed using all tested concentrations, which were non-cytotoxic. Neither the viral inhibition control (AZT) nor the solvent (DMSO) proved toxic.

### 3.7. TODB-2M and TODC-2M Compounds Inhibit Replication of HIV-1 BaL on PBMC

**Figure 8:**
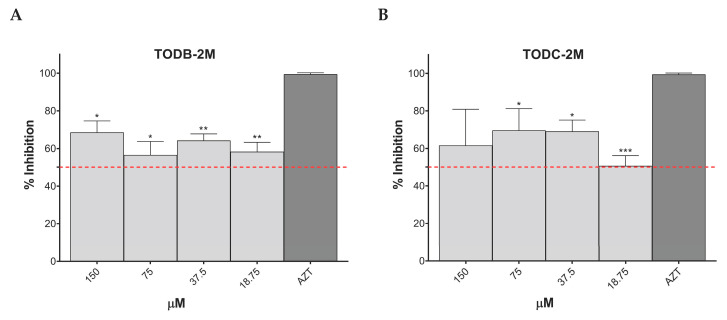

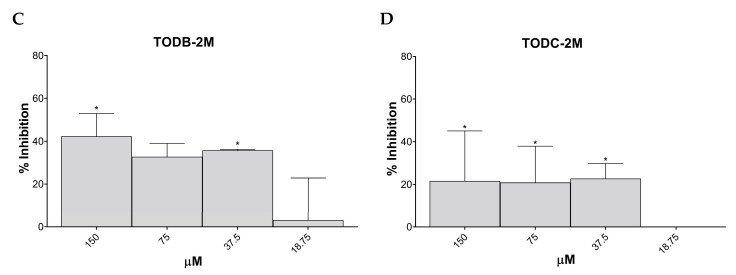
Antiviral activity of di-halogenated compounds on HIV-1 BaL replication in vitro in PBMCs. Antiviral activity was assessed using flow cytometry for intracellular p24 after TODB-2M (**A**) and TODC-2M (**B**) treatment. In addition, p24 was quantified using ELISA from the supernatant after TODB-2M (**C**) and TODC-2M (**D**) treatment. AZT (10 µM) was used as a control for inhibiting viral replication. The graphs represent the median of two independent experiments performed in triplicate but measured as a single pool; results are shown as mean ± standard deviation. Signs *, **, and *** above the columns signify *p* < 0.05, *p* < 0.01, and *p* < 0.001, respectively, and correspond to comparison with the positive inhibition control (AZT) (for (**A**,**B**)) or with the concentrations corresponding to the positive infection control (for (**C**,**D**)).

The percentage of HIV-1 BaL replication inhibition based on data obtained from flow cytometry is presented in Figure 8A,B. All concentrations of TODB-2M and TODC-2M compounds inhibited over 50% of virus replication. The positive control, AZT, inhibited 99.4% of viral replication. The minimal inhibition percentage obtained was 56.5% for TODB-2M at 75 μM and 50.6% for TODC-2M at 18.75 μM. The maximum inhibition percentage was 69.6% obtained with TODC-2M at 75 μM. These percentages showed statistically significant differences compared to the median inhibition percentages of AZT. No statistically significant differences were found for TODC-3M and YDC-3M at any tested concentrations compared to the positive inhibition control (AZT).

Viral inhibition was also demonstrated by a decrease in extracellular p24 concentration in the supernatant infected cells in the presence of increased concentrations of TODB-2M and TODC-2M compounds based on data obtained from ELISA (Figure 8C,D). The maximum inhibition percentages obtained with each treatment were 42.3% and 21.6% for TODB-2M and TODC-2M, respectively, at 150 μM. Although there were statistically significant differences between the inhibition obtained with AZT and specific concentrations of the compounds, these percentages are very low compared to AZT, which had an inhibition percentage > 90%.

### 3.8. The L-Tyrosine Derivatives Exhibit Favorable Binding Energies with the Tested Proteins

To evaluate the possible interaction of the compounds on viral proteins, in silico molecular docking of each compound with seven of the HIV-1 viral proteins—gp120 (PDB: 4J6R), gp41 (PDB: 3VTQ), RT (PDB: 4G1Q), IN (PDB: 1QS4-3OYA), protease (PDB: 5YOK), p17 (PDB: 4JMU), and p24 (PDB: 2XDE)—was evaluated.

**Figure 9:**
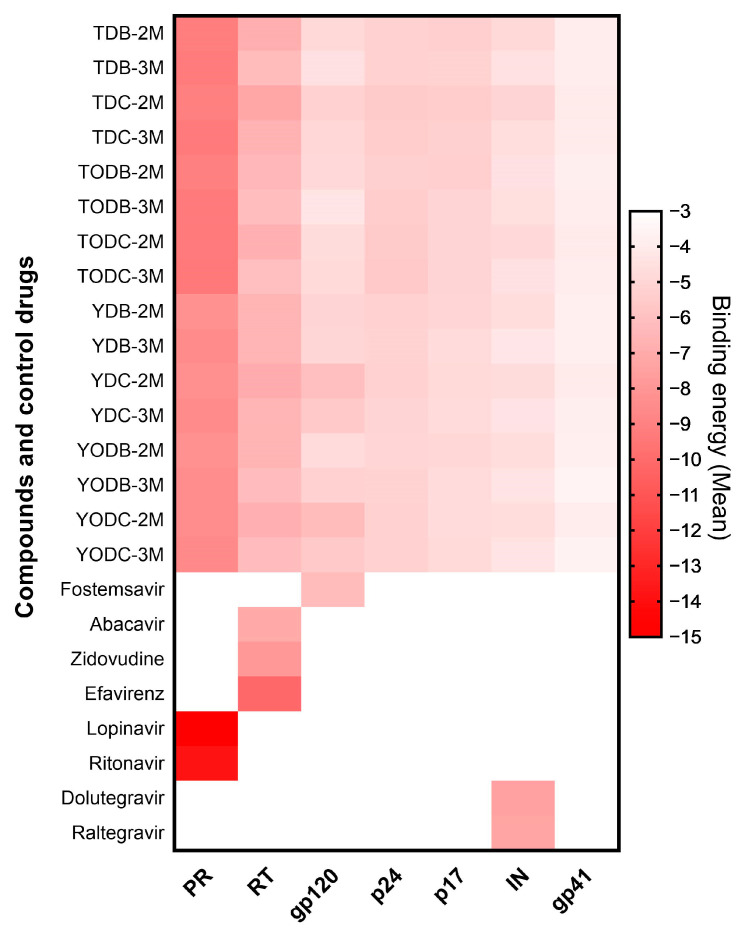
Heat map of binding energies between compounds and viral proteins and study control drugs. Free binding energies were obtained via molecular docking with AutodockVina^®^. Each interaction was analyzed in triplicate. The control drugs were only analyzed with their respective target. Less than 0 kcal/mol were considered favorable energies. The averages were graphed in red color scale. PR (protease), RT (Reverse transcriptase), gp120 (glycoprotein 120), p24 (capsid protein), p17 (matrix protein), IN (integrase), and gp41 (glycoprotein 41).

All compounds had favorable binding energies against the different proteins evaluated (between −3.53 ± 0.047 and −9.37 ± 0.09 Kcal/mol). However, only the compounds that achieved a binding energy of −6.0 Kcal/mol or lower were considered to have a strong interaction with the viral proteins. The compounds that met this threshold were those that interacted with protease and reverse transcriptase, as well as two chlorinated tertiary amines (YDC-2M and YODC-2M) that interacted with gp120 (Figure 9 and Supplementary Table S4).

To postulate a possible antiviral mechanism of the compounds that were most active against HIV-1, we used the in silico tool of molecular docking. The anti-HIV-1 effect of these compounds (TODB-2M, TODC-2M, TODC-3M, and YDC-3M) could be explained by the action on reverse transcriptase and/or viral protease, considering that the binding energies were more favorable when the compounds interacted with these proteins. Moreover, the possible mechanism of action of TODC-3M may be due to interaction with gp120.

Reverse transcriptase (RT) is a heterodimer enzyme (p66/p51) that converts viral single-stranded RNA into double-stranded proviral DNA [38]. When the complexes between the four compounds and RT were studied, it was observed that they all interacted with a large amount of the residues present in the NNRTI binding site. The binding energies obtained were between −6.73 ± 0.09 Kcal/mol and −6.1 ± 0.0 Kcal/mol. YDC-3M and TODC-2M showed the highest affinity for RT, with binding energies of −6.5 ± 0.0 Kcal/mol and −6.73 ± 0.09 Kcal/mol, respectively (Supplementary Table S4). The control drug that obtained the strongest binding energy (−10.10 ± 0.00 Kcal/mol) was efavirenz (EFV), and it interacted with residues Leu100, Lys101, Lys103, Val106, Val179, Tyr181, Tyr188, Gly190, Pro225, Phe227, Trp229, Leu234, His235, Pro 236, and Tyr318.

**Figure 10:**
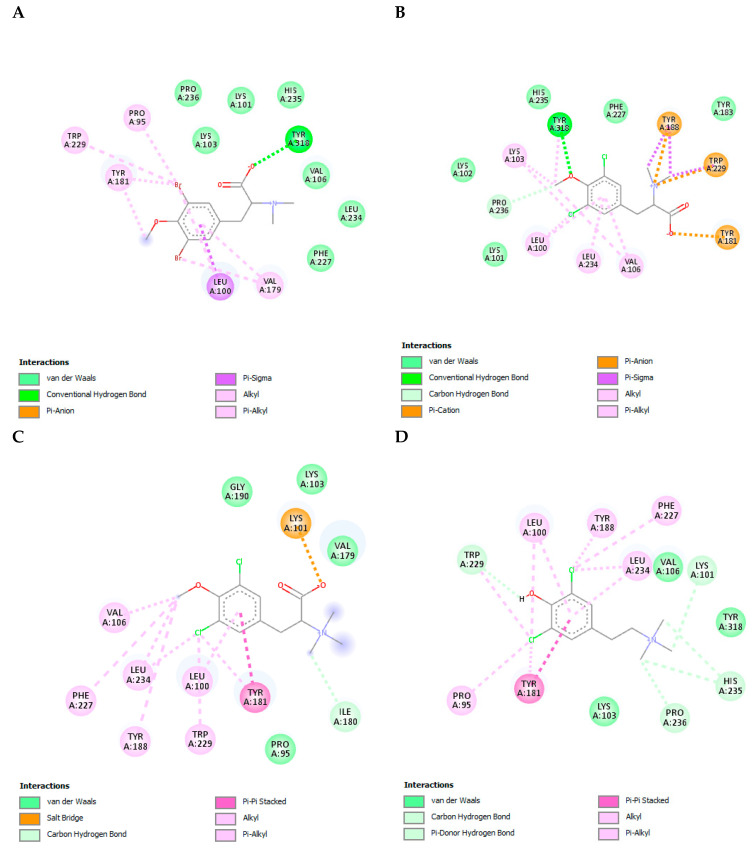
Interactions of TODB-2M, TODC-2M, TODC-3M, and YDC-3M compounds with HIV-1 reverse transcriptase. (**A**) TODB-2M. (**B**) TODC-2M. (**C**) TODC-3M. (**D**) YDC-3M. Figures obtained with Discovery Studio show the possible site of the binding of the compounds with the viral reverse transcriptase.

TODB-2M formed a hydrogen bond with Tyr318 and alkyl-type bonds with several residues, whereas TODC-2M formed a hydrogen bond with Tyr318 and several pi-type bonds with other residues present in the NNRTI binding site (Figure 10A,B). TODC-3M formed Van der Waals-type interactions with several residues and a salt bridge with Lys101. However, tyramine YDC-3M formed a non-covalent Pi–Pi-type interaction with Tyr181 and several alkyl and Van der Waals-type interactions with other residues present in the NNRTI binding site (Figure 10C,D). These interactions are described as polar or non-polar in Supplementary Table S5 and are shown in Supplementary Figure S17.

HIV protease is a key enzyme in the viral replication process, as it is responsible for the maturation of the viral particles. It is an aspartyl protease consisting of two monomers of 99 amino acids. The aspartyl protease motif (catalytic triad: Asp25-Thr26-Gly27) is a key target for drug development [39,40]. Compounds TODB-2M, TODC-2M, TODC-3M, and YDC-3M obtained binding energies between −9.37± 0.09 Kcal/mol and −8.5 ± 0.0 Kcal/mol when interacting with viral PR (Supplementary Table S4). The control drug that obtained the strongest binding energy (−14.97 ± 0.05 Kcal/mol) was lopinavir (LPV) and interacted with residues Asp25, Asp25′, Ile50, Ile50′, Gly27, and Asp29, forming six hydrogen bonds, and with other residues of both chains, close to the active site by Van der Waals interactions. It was observed that our compounds interact directly with the key amino acids in the active site.

**Figure 11:**
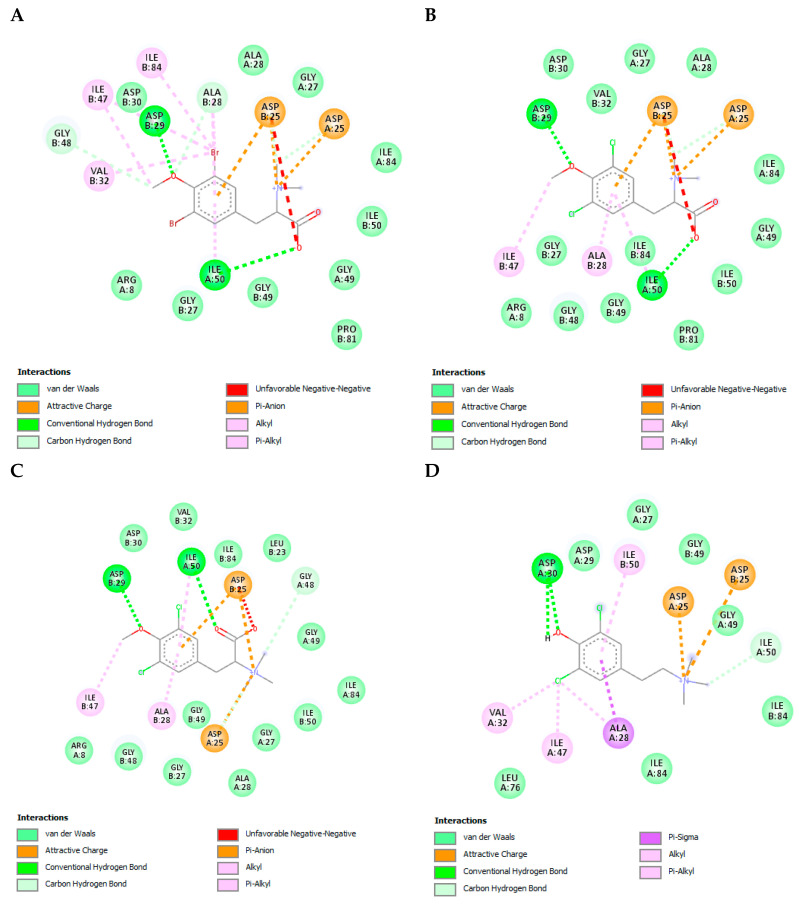
Interactions of TODB-2M, TODC-2M, TODC-3M, and YDC-3M compounds with HIV-1 protease. (**A**) TODB-2M. (**B**) TODC-2M, (**C**) TODC-3M. (**D**) YDC-3M. Figures obtained with Discovery Studio show the possible site of the binding of the compounds with the viral protease.

Each compound interacts with specific residues in the protease active site through various types of bonds and interactions, including hydrogen bonds, salt bonds, carbon-hydrogen bonds, alkyl-type bonds, Van der Waals interactions, and Pi-sigma bonds with other residues near the active site (Figure 11). Among the most prominent interactions of the TODB-2M, TODC-2M, and TODC-3M compounds is the formation of hydrogen bridges with the oxygen in the ortho position of the phenolic ring and Asp29′, with the carboxyl group and Ile50 as well. In contrast, YDC-3M interacts by forming two hydrogen bonds with Asp30. Similarly, all compounds form salt bonds between Asp25 and Asp25′ and the amino group (Figure 11A–D). These interactions are described as polar or non-polar in Supplementary Table S5 and are shown in Supplementary Figure S18.

**Figure 12:**
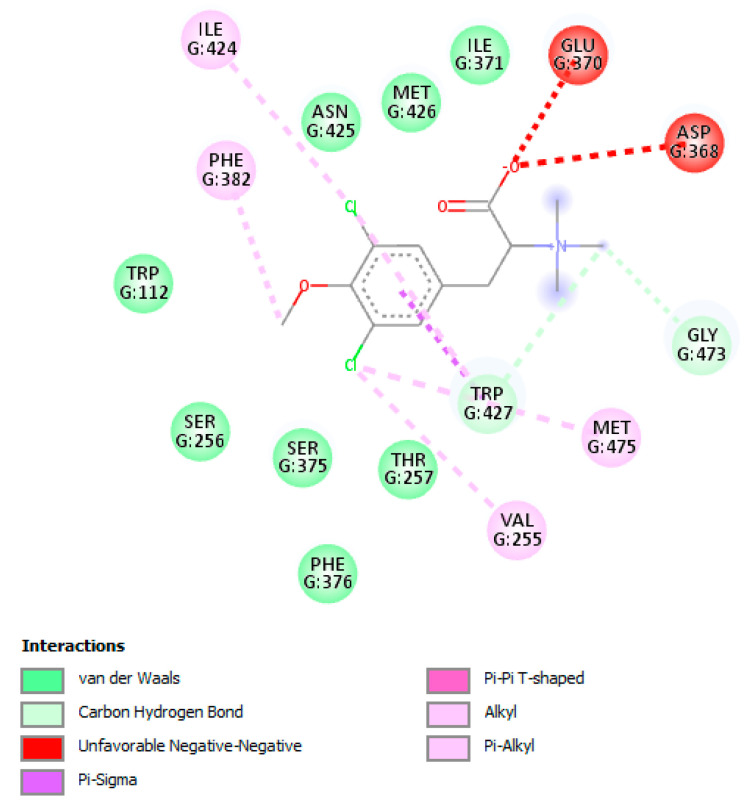
Interactions of TODC-3M compound with HIV-1 gp120. Figure obtained with Discovery Studio shows the possible site of the binding of the compounds with the viral gp120.

Gp120 is a viral surface protein responsible for virus entry. It has five variables (V1–V5) and five constant (C1–C5) domains. The V3 loop is important for membrane fusion and co-receptor specificity, that is, the use of CCR5 versus CXCR4 [41]. The approved control drug inhibiting this protein is fostemsavir (FTR), which obtained binding energy of −6.23 ± 0.40 Kcal/mol and formed hydrogen bond interactions with Asn425, Gly431, and Gln432 and hydrophobic interactions with residues Thr257, Asp368, Glu370, Ile371, Met426, Trp427, Ala430, Gly473, and Met475. The TODC-3M value (ΔGbind = −4.83 ± 0.24 Kcal/mol) formed Van der Waals interactions with the Trp112 residues, Val255, Ser256, Thr257, Ile371, Ser375, Phe376, Phe382, Ile424, Asn425, Met426, Trp427, Gly473, and Met475. In addition, this compound presented an unfavorable negative–negative interaction between the carboxyl group and Asp368 and Glu370 (Figure 12 and Supplementary Table S4). These interactions are described as polar or non-polar in Supplementary Table S5 and are shown in Supplementary Figure S19.

### 3.9. The L-Tyrosine Derivatives Exhibit Stable Interaction with the Tested Proteins during Molecular Dynamics Simulations

To assess the stability of the RT and PR with TODC-2M and TODB-2M, a series of molecular dynamic simulations was performed. We monitored the RMSD of the proteins and ligands, AutoDock Vina score, and the number of hydrogens bonds throughout 100 ns trajectories.

**Figure 13:**
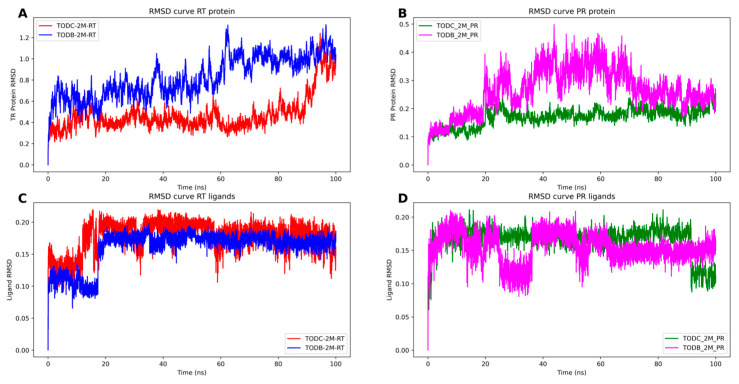
Molecular dynamics trajectories for PR and RT proteins bond to TODC-2M and TODB-2M ligands. (**A**) RMSD curve of the TR protein bond to ligands TODC-2M (red) and TODB-2M (blue). (**B**) RMSD curve of the PR protein bond to ligands TODC-2M (green) and TODB-2M (magenta). (**C**) RMSD curve of the ligands TODC-2M (red) and TODB-2M (blue) bound to RT protein. (**D**) RMSD curve of the ligands TODC-2M (green) and TODB-2M (magenta) bound to PR protein.

**Figure 14:**
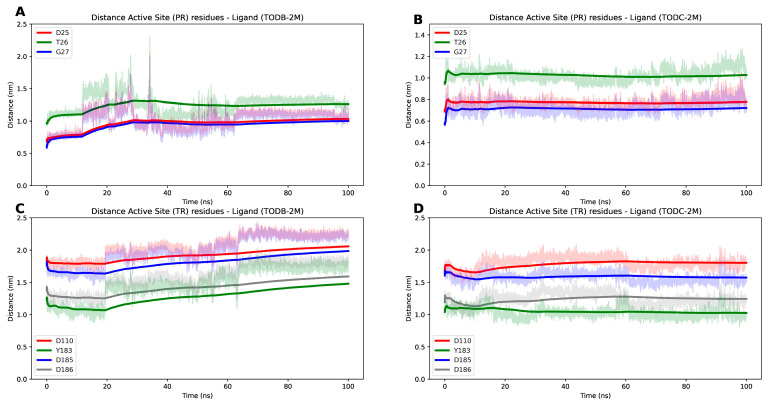
Distance plot between active site residues and ligands through the 100 ns simulation for RT and PR protein bonds to TODB-2M and TODC-2M ligands. (**A**) TODB-2M and active site residues from PR protein, Asp25 (red), Thr26 (green), and Gly27 (blue). (**B**) TODC-2M and active site residues from PR protein, Asp25 (red), Thr26 (green), and Gly27 (blue). (**C**) TODB-2M and active site residues from TR protein, Asp110 (red), Tyr183 (green), Asp185 (grey), and Asp186 (blue). (**D**) TODC-2M and active site residues from TR protein, Asp110 (red), Tyr183 (green), Asp185 (grey), and Asp186 (blue).

Stable simulations were observed for the complexes, with ligand RMSD below 0.3 nm and protein RMSD below 1.3 nm (Figure 13). The RT protein exhibited fluctuation due to its flexible regions, while the interaction between the monomers of the protease protein showed some flexibility in the presence of the ligand TODB-2M between 20 to 70 ns of the simulation. However, both proteins demonstrated stable interactions with the docked ligands over the 100 ns simulation. This stability is evident as the distance between the ligands and active site residues of PR and TR (Figure 14) did not show significant changes. Remarkably, the protease–TODC-2M complex exhibited the most stable interaction (Figure 14B). Overall, analysis of the complexes after 100 ns indicated that the hydrogen bonds predicted during the docking phase were largely maintained (Figure 14). Regarding the interaction between TODB-2M and both proteins, hydrogen bonds appeared weak during approximately 50% of the simulation, but after 60 ns, they began to rearrange their conformation. TODC-2M exhibited stronger hydrogen bonds throughout the simulation, showing overall more significant interaction than TODB-2M.

**Figure 15:**
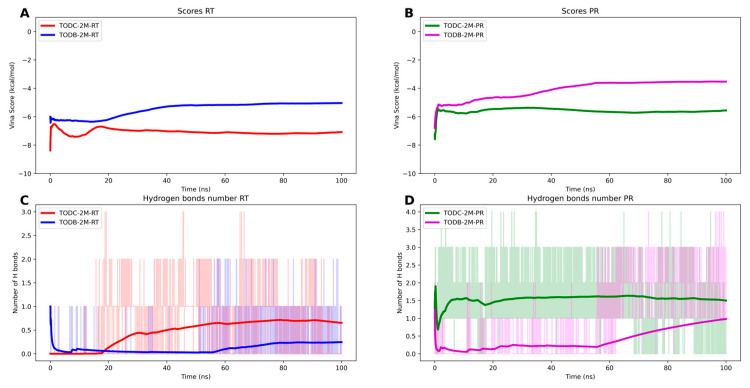
Evolution of the score vina and number of hydrogen bonds through the 100 ns simulation for RT and PR protein bonds to TODC-2M and TODB-2M ligands. (**A**) TODC-2M (red) and TODB-2M (blue) with TR protein. (**B**) TODC-2M (green) and TODB-2M (magenta) with PR protein. (**C**) TODC-2M (red) and TODB-2M (blue) with TR protein. (**D**) TODC-2M (green) and TODB-2M (magenta) with PR protein.

Finally, evaluation of the Vina scores during the simulation (Figure 15) revealed that TODC-2M exhibited a better average affinity toward both targets than TODB-2M. In the case of the interaction with the protease, we observed a decrease compared to the initial docking result, possibly due to the flexibility of the protein in the region where the compounds interact. This region of the dimer interaction exhibits high flexibility due to the loops that form the interaction site. We employed the relaxed complex scheme approach [42] for this analysis, using frames obtained from the molecular dynamics simulations to calculate the cumulative average score using the AutoDock Vina function [32].

## 4. Discussion

HIV/AIDS continues to be a public health problem worldwide. Despite the existence of combined treatment that reduces the circulating viral load to undetectable levels, there are problems of toxicity and resistance to the currently used drugs [43]; therefore, the search for new antiviral strategies continues.

In this study, we evaluated 16 synthetic di-halogenated compounds derived from L-tyrosine, which are structural derivatives of compounds previously isolated from the marine sponges *Verongula rigida* and *Aiolochroia crassa* [14].

The in vitro cytotoxicity of our compounds was initially evaluated in TZM-bl cells. Cell viability higher than 77% at a concentration of 150 µM for all compounds except YDB-3M was found (Figure 2). These data are congruent with those reported for other halogenated L-tyrosines, where in vitro cytotoxicity lower than 25% was reported in the U373-MAGI cell line treated with concentrations ≤ 320 µM [14]. Similarly, our collaborators found cell viability of VERO cells up to 90% when treated with L-tyrosine-derivate compounds with free phenolic OH at a concentration of 250 µM [44]. With these results, we calculated the CC_50_ for each compound, where we ruled out a cytotoxic effect as a potential mechanism to explain the viral inhibition.

The discovery and development of a new drug is a costly and time-consuming process; therefore, in recent years, ADMET technology has been adopted to predict the pharmacological potential of a compound and thus avoid failure [45]. The compounds presented in our study have low and medium cumulative toxicity, like the control drugs evaluated (Table 2). Only the compounds in YODB-2M, YODB-3M, YODC-2, and YODC-3M were predicted to be toxic because they had a neurotoxic effect by inducing phospholipids and a cardiac effect by blocking hERG channels. The results of toxicity prediction of reference compounds are contrastable with actual data. Among the adverse effects of protease inhibitors such as lopinavir and ritonavir, hepatotoxicity has been reported, similar to that observed in our in silico prediction with increased liver enzymes. Likewise, drugs such as efavirenz cause cutaneous hypersensitivity and neurotoxicity, effects also observed in the in silico analysis [46]. In the ADMET_Risk variable, which serves as a comprehensive filter that includes 24 parameters addressing absorption, metabolism, toxicity, and pharmacokinetic parameters associated with the five rules of Lipinski [47], all our compounds obtained a minimal overall risk as they had a score below 7, and they were even improved in some respect than the control drugs lopinavir and ritonavir with scores of 7.5 and 9.7, respectively (Table 2). The compounds evaluated in the study satisfied Lipinski’s rule of five (Supplementary Table S1); therefore, di-halogenated compounds with potent antiviral activity (Supplementary Tables S2 and S3) would be appropriate candidates as oral drugs.

Other compounds derived from L-tyrosine structurally related to ours have been previously reported for their antiviral activity against HIV-1 [14,48,49], respiratory syncytial virus [50], and SARS-CoV-2 [51,52]. In contrast, in our study, the compounds presented variable activity to inhibit the HIV-1 infection, depending on the strain evaluated and the structural modifications of each compound.

We included an antiviral screening strategy to evaluate the antiviral effect of the 16 compounds against HIV-1 infection. The HIV-1 BaL (R5) strain was used as a viral model since this virus is predominantly detected during early infection. In the natural course of infection, individuals become infected with this strain, but in the more chronic stages of infection, a change of tropism occurs [53]. The tropism shift from R5 to X4 strain is associated with accelerated CD4+ T cell depletion and disease progression and is primarily explained by modifications in the region of gp120 that encode the V3 loop [54]. The HIV-1IIIB (X4) strain was used to evaluate this model. In addition, HIV-GFP-VSV-G, which does not have the HIV envelope, but instead uses the vesicular stomatitis virus envelope for entry, was used as a model to evaluate the inhibition of the replicative cycle after entry. This, coupled with the HIV-GFP-VSV-G pseudotyped virus assays, will allow us to determine whether the compounds act by inhibiting early or late stages of infection and thus determine the possible mechanism of action involved in the antiviral activity.

The brominated compounds TDB-2M and TDB-3M did not inhibit the replicative activity of the strains tested. In contrast, the chlorinated compounds TDC-2M and TDC-3M showed antiviral activity in the R5 strain, while no antiviral activity in the X4 strain was observed (Supplementary Tables S2 and S3). These compounds were also evaluated by colleagues against other viral models; it was observed that all of them increased the production of infectious Dengue virus -2 particles but showed a potential to significantly inhibit the production of infectious viral particles of the Chikungunya virus, with inhibition percentages between 60.1% and 71.5% [44]. That suggests that the antiviral activity of these compounds depends on the viral model evaluated and that the inhibition of HIV replicative activity is not affected by exposure to brominated L-tyrosine derivates with free phenolic OH compounds, probably due to a factor inherent to the viral infection.

For methyl ether L-tyrosine-derivate compounds, TODB-2M inhibited strains R5 and X4, while its respective quaternary ammonium salt (TODB-3M) showed no activity against any of the strains tested. TODC-2M inhibited replication of strain R5 and strain X4. In addition, TODC-3M effectively inhibited replication of strain R5 (Supplementary Tables S2 and S3). To date, there are no other studies evaluating the antiviral activity of these methyl ether L-tyrosine-derivate compounds in this viral model or in others, but several authors have evaluated the activity of bromotyrosines derived from marine sponges such as mololipids [49], psammaplysine D [55], aeroplysinin-1 [14], fistularin-3 [56], and purealidin B [14] and observed antiviral activity against different evaluated HIV-1 strains (e.g., Haitian RF strain of HIV-1, HIV-GFP-VSV-G virus), attributing their activity to the inhibition of different steps of the HIV replicative cycle. This can be extrapolated with this study due to the structural similarity with our compounds since all of them are tyrosine derivatives and present di-halogenation with Br in the structure.

The structural difference between TDB-2M and TODB-2M is the addition of a methyl group on the free phenolic OH. Interestingly, this structural difference appears to favor the antiviral activity of the compound, such as was observed for TDC-3M and TODC-3M. This opens the door to new studies in which modifications such as the addition of methyl groups in compounds with antiviral potential could enhance their activity as potential therapeutic agents against HIV.

In tyramine-derivate compounds, YDB-2M had no antiviral activity against strains R5 or X4. The quaternary ammonium salt YDB-3M inhibited replication of strain X4 at the lowest concentration tested but had no activity against strain R5. At the highest concentration evaluated, the compounds YDB-2M and YDB-3M may produce an increase in the strains R5 HIV-1 replication (Supplementary Tables S2 and S3). This effect may be due to the action of the compound on TZM-bl cells, increasing cell proliferation, enhancing cell metabolism or gene transcription, and consequently increasing cellular and viral proteins synthesis. Our collaborators observed a similar effect in the Zika viral model, with di-halogenated compounds with chlorine and bromine structurally like ours [44]. However, further studies are needed to elucidate how viral replication is enhanced. The chlorinated analogue YDC-2M also had no activity against the strains tested, while YDC-3M inhibited replication of both strains. Unexpectedly, an inverse relationship was observed between YDC-3M concentration and the anti-HIV-1 activity. This means that the inhibitory activity decreases as the YDC-3M concentration increases. Higher concentrations of the compound are not optimal for achieving its antiviral activity. At these concentrations the compounds may produce saturation of TZM-bl cells, preventing the entry of the compound or the interaction with viral proteins, which may be detrimental to the evaluation of the antiviral effect. Finally, methyl ether tyramine-derivate compounds were the least active against the virus, as only YODB-3M had anti-HIV activity, inhibiting the replication of strain X4. At 75 µM, the compound YODB-2M increased the replication of HIV-1 strains R5 and X4 (Supplementary Tables S2 and S3). As with the brominated analogues of tyramine derivates with free phenolic OH compounds, this effect may be due to the action of the compounds on the cells, increasing cell proliferation, enhancing cell metabolism or gene transcription, and consequently increasing cellular and viral proteins synthesis. Other compounds derived from bromotyrosines have exhibited variable ability to inhibit HIV-1 replication in vitro, among these are YDB-3M and YODB-3M. The latter inhibited HIV-1 entry (X4 strain) in a dose-dependent manner, but neither compound had activity against the R5 strain [14]. These results are congruent with those found by us.

Following the antiviral screening, four compounds with the highest inhibitory capacity against viral strains were selected and evaluated against a pseudotyped virus (HIV-GFP-VSV-G). TODB-2M, TODC-2M, and YDC-3M compounds obtained an inhibition percentage between 12.5–44.2% (Figure 6). Similarly, these four compounds were chosen to evaluate their anti-HIV activity in a different cell model. For this purpose, we used PBMCs since it is a biological model more representative of the in vivo environment and can better mimic the natural infection process compared to cell lines. Upon infecting these cells with HIV-1 BaL and treating them with TODB-2M and TODC-2M at 150 µM, we observed an inhibition percentage of viral replication greater than 60% and 20% (Figure 8), based on the quantification of intracellular and extracellular p24, quantified using flow cytometry or ELISA, respectively. There were some differences between the inhibition percentages obtained using flow cytometry and ELISA, which was expected since both techniques measure different products produced during the viral cycle. In this case, the amount of p24 released from the cells was lower than that found intracellularly. However, most of the concentrations evaluated with both techniques showed a statistically significant inhibitory effect when compared to the positive inhibition control. Likewise, these compounds had an antiviral effect when evaluated with R5 and X4 strains in TZM-bl cells; this suggests that the mechanism of action of these compounds may be due to interactions with viral proteins involved in the replication steps that occur after virus entry. This mechanism, coupled with in silico assays, may indicate that inhibition of viral replication is due to interaction with protease or RT. The TODC-3M compound did not exhibit antiviral activity using HIV-GFP-VSV-G, as with the X4 strain. This suggests that the possible mechanism of action is at virus entry, by interacting with gp120 of the R5 strain. Wu ZY et al., observed that di-brominated compounds, such as diarylpyridinamine analogues (DAPA), favored antiviral activity over di-chlorinated compounds against wild-type HIV-1 NL4-3 (X4 strain) infection in TZM-bl cells [57]. These results are consistent with ours when using the X4 strain, where it was observed that three of the five compounds exhibiting antiviral activity were di-brominated. In contrast, higher antiviral activity was observed when the di-chlorinated compounds were used against the R5 strain.

Interestingly, by using HIV-GFP-VSV-G as a viral model, the TODB-2M compound showed the highest percentage of inhibition; this compound has two Br halogens in its structure, while the activity of di-chlorinated compounds was significantly lower. Our study suggests that these compounds could be a promisor to evaluate in studies as potential anti-HIV-1 drugs.

Although the concentrations at which the di-halogenated compounds had an antiviral effect are higher than the AZT concentration, it is important to consider that this drug is potentially toxic [58]. In addition, although the compound concentrations with antiviral activity were not toxic in the cells evaluated, it is important to carry out in vivo studies to determine whether these concentrations are safe. Therefore, searching for new molecules with a safer pharmacological profile is necessary.

Molecular docking is a frequently used methodology for designing and discovering new bioactive molecules due to the potential to simulate interactions between a ligand and a target and predict the binding mode and affinity between them, thus predicting whether it can produce the expected biological effect [59,60]. Molecular docking methods can be a helpful screening tool for predicting ligand–protein interactions. Still, it is crucial to consider the limitations and potential sources of error when interpreting the results, as they may only sometimes be accurate. For example, HIV protease is a flexible protein whose conformation can change depending on the ligand to which it is bound [61]. This flexibility can make it difficult to predict the binding mode of a ligand using docking methods; therefore, false positives may exist. For this reason, it is important to remember that molecular docking is an approximation method, and further studies are needed to corroborate this prediction.

In our study, all 16 compounds interacted with viral RT and PR. In addition, compounds YDC-2M and YODC-2M complied with the threshold when interacting with gp120 (Supplementary Table S4 and Figure S9). In this case, we observed a higher affinity score for compounds with Cl in their structure, which is congruent with that found by Bollini et al., who observed that chlorine substitution in the terminal phenolic ring predicts more favorable binding energies with RT [62]. The anti-HIV-1 effect of TODB-2M, TODC-2M, and YDC-3M compounds could be attributed to action on viral RT and/or PR, since the binding energies were more favorable upon interaction with these proteins. The TODC-3M compound, in addition to interacting with these proteins, action may be due to inhibition of entry by interacting with gp120.

RT is an enzyme that converts viral single-stranded RNA into double-stranded proviral DNA [63]. The results presented in this work agree with those obtained by Ortega et al. when evaluating the antiviral activity of myricetin derivatives as NNRTIs against HIV-1IIIB (strain X4) because the binding interactions of our compounds also occurred with residues of the hydrophobic NNRTI-binding pocket (Figure 10), specifically with residues Leu100, Lys101, Lys103, Val106, Val179, Tyr181, Tyr188, Phe227, Trp229, and Tyr318 of p66 subunit [64]. Likewise, it has been described that nevirapine (FDA-approved drug as an NNRTI) acts against RT by binding to this hydrophobic pocket specifically [65,66]. Therefore, our compounds TODB-2M, TODC-2M, and YDC-3M could be working as NNRTIs.

HIV protease is responsible for the maturation of the viral particles. The protease active site is formed by the catalytic triad: Asp25-Thr26-Gly27, this region is a crucial target for drug development [39,40]. Our study observed that TODB-2M, TODC-2M, and YDC-3M compounds interact directly with the critical amino acids in the active site (Figure 11); these interactions could enhance the inhibitory effect by blocking the structure in its closed conformation and thus hindering the entry of natural substrates.

Gp120 is the viral protein that mediates entry into the host cell by interacting with the CD4 receptor and a coreceptor (CCR5 or CXCR4) [67]. The drug fostemsavir binds to this protein and interacts with residues Asp425, Met426 and Trp427 and alters their conformation, blocking subsequent binding to the CD4 receptor [68]. Our study predicted that the compound TODC-3M interacted with these crucial residues in the protein binding to the receptor (Figure 12), indicating a similar effect to fostemsavir, blocking entry.

To examine the stability of the complex with the highest docking score, we conducted 100 ns of molecular dynamics simulations. Both proteins exhibited some degree of flexibility, but overall, their structures remained stable, as reported in previous simulations of these proteins [69,70]. Prior studies have analyzed the interactions of these proteins using molecular dynamics to investigate their stability with different drugs [71,72,73]. Our study observed that the TODC-2M and TODB-2M ligands maintained stable interactions with both proteins throughout the simulations. TODC-2M yielded superior results, with higher retention of hydrogen bonds and a better AutoDock Vina score.

For HIV-1 protease, previous studies have emphasized the importance of protein flaps in substrate or inhibitor interactions, given their dynamic nature of opening and closing the active site [74]. An effective inhibitor for the protease would be able to lock the hinge and prevent the flaps from opening [72,74]. Our complexes with both ligands demonstrated that the flaps remained closed throughout the simulation, which is crucial to avoid interactions with the natural substrate.

In our study, 16 compounds were evaluated and modified with bromine and chlorine halogens. The use of halogenated compounds can give an advantage for the design of new drugs due to the formation of halogen bonds (X) formed by chlorine (Cl), bromine (Br), and iodine (I), which are comparable to hydrogen bonds and can increase the protein-ligand affinity [75,76]. Previous studies have shown that the presence of Br in the molecules improves the selectivity of protein–ligand interactions, while the presence of Cl improves the pharmacokinetic properties [77]. The results obtained in this study open the door to further investigations of di-halogenated compounds as potential lead compounds; that is, molecules or chemical compounds that can be optimized from the initial molecule to improve the activity, potency, and selectivity of the compound to progress toward the development of antiviral agents.

## 5. Conclusions

TODB-2M, TODC-2M, TODC-3M, and YDC-3M are new synthetic di-halogenated compounds derived from L-tyrosine that could be considered as lead compounds to develop a promising anti-HIV-1 agent. According to biological activity assays, TODB-2M, TODC-2M, and YDC-3M compounds showed inhibitory activity against HIV-1 replication viruses with CCR5 and CXCR4 tropism and a pseudotyped virus, while TODC-3M was effective against CCR5 tropic virus. These compounds demonstrated antiviral activity, but the mechanism of action has not yet been elucidated experimentally; however, based on the viral models used in our study, we can hypothesize that TODB-2M, TODC-2M, and YDC-3M act by inhibiting steps downstream of viral entry, while TODC-3M would inhibit steps upstream of entry. According to in silico results, the possible mechanism is the interaction with reverse transcriptase and/or viral protease or with the envelope protein in the case of TODC-3M. In addition, the in vitro and in silico methodology used in this study are valuable tools for studying antivirals, determining possible mechanisms of action and toxic effects associated with the chemical nature of different compounds. The results obtained in this study underline the importance of further research and evaluation of the different stages of the virus cycle, such as viral integration, protein translation, and assembly, especially to unravel the mechanisms of action of these compounds. Finally, this result allows further research on di-halogenated compounds as potential lead compounds.

## Figures and Tables

**Table 1:** Viral proteins and binding sites are defined in the study as targets for molecular docking with the compounds. The selected proteins were obtained from the Protein Data bank (PDB) by X-ray diffraction method. The chains were selected based on the presence of the active site sequence.

Protein	PDB Code	Resolution (Å)	Sequence Length	Chain	Active Site Amino Acids
gp120	4J6R	1.64	359	A	Trp112, Thr257, Asp368, Glu370, Ser375, Phe382, Ile424, Met426, Trp427, Met434, Met475
gp41	3VTQ	1.53	37	A	Leu565, Leu566, Thr569, Val570, Trp571, Gly572, Ile573, Lys574, Leu576, Gln577
RT	4G1Q	1.51	557	A	Asp110, Tyr183, Asp185, Asp186, Leu100, Lys101, Lys103, Val106, Thr107, Val108, Val179, Tyr181, Tyr188, Val189, Gly190, Phe227, Tyr318, Glu138
Protease	5YOK	0.85	100	A-B	Asp25, Thr26, Gly27
Integrase	1QS4	2.1	154	A	Asp64, Asp116, Glu152
p24	2XDE	1.4	145	A	Phe32, Glu35, Val36, Asn57, Val59, Glu60, His62, Gln63, Ala65, Gln67, Lys70, Thr107, Met118, Thr119
p17	4JMU	2.0	112	A	Leu21, Arg22, Pro23, Tyr29, Lys32, His33, Trp36, Ser77, Leu88, Asn80, Thr81, Thr81, Thr97, Lys98, Leu101

**Table 2:** In silico toxicity score of ADMET properties. ALP: Alkaline Phosphatase, GGT: Gamma-glutamyl Transferase, LDH: Lactate Dehydrogenase, SGOT: Aspartate Aminotransferase, SGPT: Alanine Aminotransferase, ABC: Abacavir, DTG: Dolutegravir, EFV: Efavirenz, FTR: Fostemsavir, LPV: Lopinavir, RAL: Raltegravir, RTV: Ritonavir, and AZT: Zidovudine.

	TDB-2M	TDB-3M	TDC-2M	TDC-3M	TODB-2M	TODB-3M	TODC-2M	TODC-3M	YDB-2M	YDB-3M	YDC-2M	YDC-3M	YODB-2M	YODB-3M	YODC-2M	YODC-3M	ABC	DTG	EFV	FTR	LPV	RAL	RTV	AZT
Chromosome aberrations	0	1	0	1	0	1	0	1	1	1	1	1	0	0	1	0	1	0	0	0	0	0	0	1
Skin sensitization	1	1	0	0	1	1	0	0	1	1	1	0	1	1	0	0	1	1	1	0	0	1	0	1
Respiratory sensitization	0	0	0	0	1	0	1	0	1	1	1	1	1	1	1	1	1	0	1	0	0	0	0	0
Neurotoxicity	0	0	0	0	0	0	0	0	0	0	0	0	1	1	1	1	0	0	1	0	0	0	0	0
Cardiac toxicity	0	0	0	0	0	0	0	0	0	0	0	0	1	1	1	1	0	0	0	0	0	0	0	0
Estrogen receptor tox	1	1	1	1	1	1	1	1	1	1	1	1	0	0	0	0	0	0	0	1	0	0	0	0
Androgen receptor tox	0	0	0	0	1	1	1	1	1	1	1	1	0	1	0	1	0	0	1	0	1	0	1	0
ALP increase	0	0	0	0	0	0	0	0	0	0	0	1	0	0	0	0	1	0	1	0	1	0	1	1
GGT increase	1	0	0	0	1	0	0	0	1	0	0	0	1	0	0	0	0	0	0	0	1	0	1	1
LDH increase	0	0	0	0	0	0	0	0	0	0	1	1	1	1	0	0	0	0	0	0	1	0	1	1
SGOT increase	0	0	0	0	0	0	0	0	0	0	0	0	0	0	0	0	1	1	1	0	0	1	0	1
SGPT increase	0	0	0	0	0	0	0	0	0	0	0	0	0	0	0	0	1	0	1	0	1	0	1	1
Reproductive tox	0	0	0	0	0	0	0	0	1	0	0	0	0	0	0	0	0	0	1	0	1	0	1	1
Accumulated tox	3	3	1	2	5	4	3	3	7	5	6	6	6	6	4	4	6	2	8	1	6	2	6	8
ADMET_Risk	0.0	0.9	0.0	1.0	0.0	0.7	0.9	1.4	0.0	0.3	2.0	2.8	0.5	0.4	2.3	1.8	1.4	0.0	4.7	5.0	7.5	2.5	9.7	5.4

**Table 3:** Selectivity index of active compounds against HIV-1 BaL. NA: Not applicable; values cannot be calculated because exact IC50 data are unavailable.

Compound	CC_50_ (µM)	IC_50_ (µM)	SI
TDC-2M	~174.4	140.7	~1.24
TDC-3M	>300	NA	NA
TODB-2M	164.9	113.5	1.45
TODC-2M	>300	67.42	>4.45
TODC-3M	74.9	NA	NA
YDC-3M	39.88	37.7	1.07

**Table 4:** Selectivity index of active compounds against HIV-1_IIIB_. NA: not applicable; values cannot be calculated because exact IC50 data are unavailable.

Compound	CC_50_ (µM)	IC_50_ (µM)	SI
TODB-2M	164.9	~82.68	~1.99
TODC-2M	>300	~39.15	~7.66
YDB-3M	~100.9	3.09	~32.65
YDC-3M	39.88	22.31	1.65
YODB-3M	78.7	NA	NA

**Table 5:** Selectivity index of the compounds against HIV-GFP-VSV-G. NA: not applicable; values cannot be calculated because exact IC50 data are unavailable.

Compound	CC_50_ (µM)	IC_50_ (µM)	SI
TODB-2M	164.9	141.8	1.16
TODC-2M	>300	NA	NA
YDC-3M	39.88	NA	NA

## Data Availability

The data presented in this study are available on reasonable request from the corresponding author.

## Supplementary Materials

The following supporting information can be downloaded at https://www.mdpi.com/article/10.3390/cimb45100516/s1, Supplementary Figures S1–S16: 1H NMR spectra of the evaluated di-halogenated compounds. Table S1: di-halogenated compounds evaluated in the study satisfied Lipinski’s rule of five. Table S2: percentage inhibition of HIV-1 BaL replication of each di-halogenated compound; Table S3: percentage inhibition of HIV-1IIIB (X4 strain) replication of each di-halogenated compound; Table S4: binding energies between compounds and viral proteins and study control drugs; and Table S5: interactions of the promising compounds with RT/PR/gp120. Supplementary Figure S17: Binding of di-halogenated compounds to the active site region of reverse transcriptase. Supplementary Figure S18: Binding of di-halogenated compounds to the active site region of protease. Figure S19. Binding of di-halogenated compounds to the active site region of gp120.
