# Supplementary material for: In Vitro and In Silico Antiviral Activity of Di-Halogenated Compounds Derived from L-Tyrosine against Human Immunodeficiency Virus 1 (HIV-1)
Source: Curr Issues Mol Biol. 2023 Oct 9;45(10):8173–200. doi: 10.3390/cimb45100516 (PMC10605077; doi:10.3390/cimb45100516)
Supplement: Supplementary file 1 [file cimb-45-00516-s001.zip › cimb-2549046-supplementary.pdf]

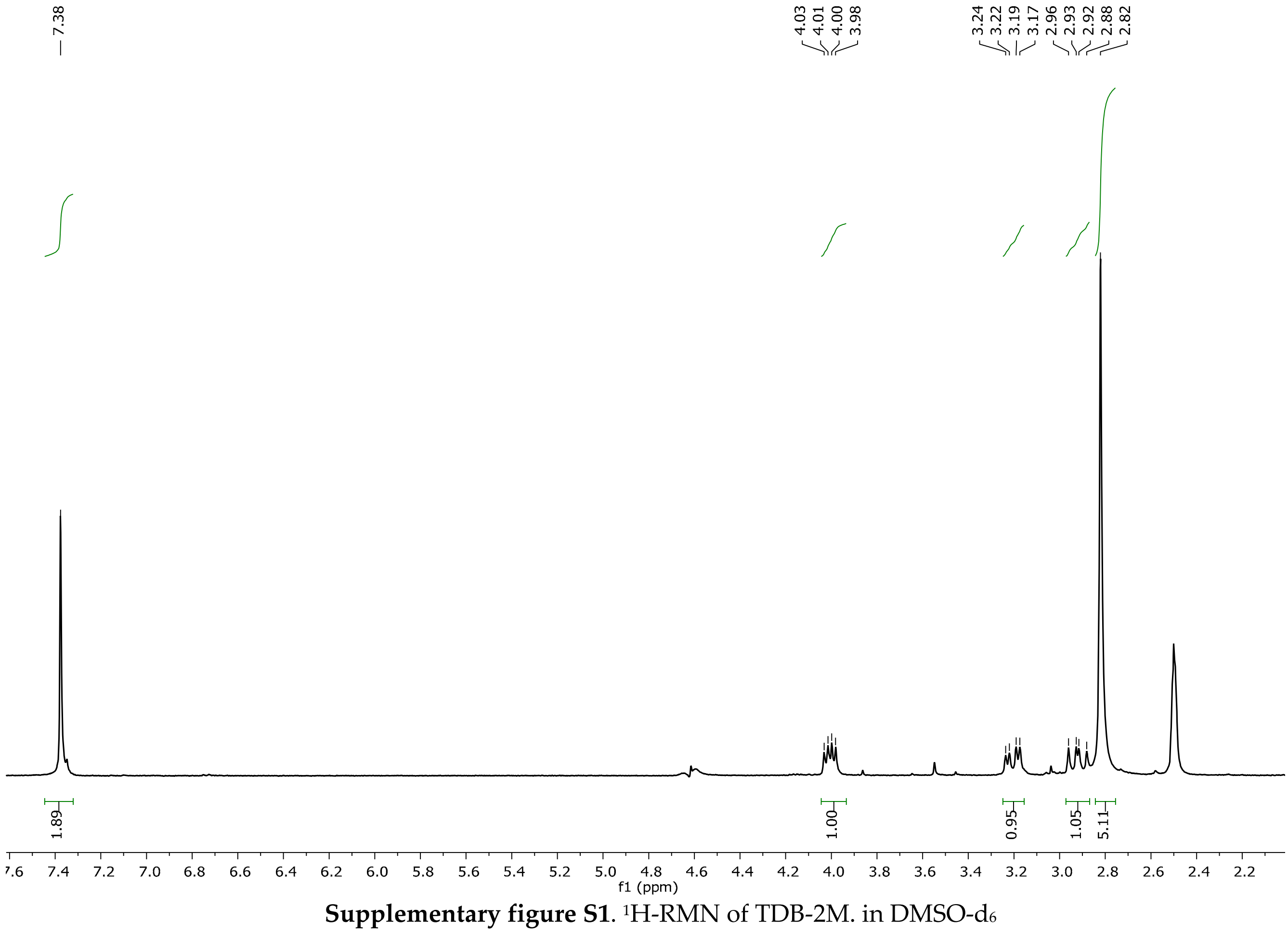

Supplementary figure S1. ^{1}H-RMN of TDB-2M. in DMSO-d_6

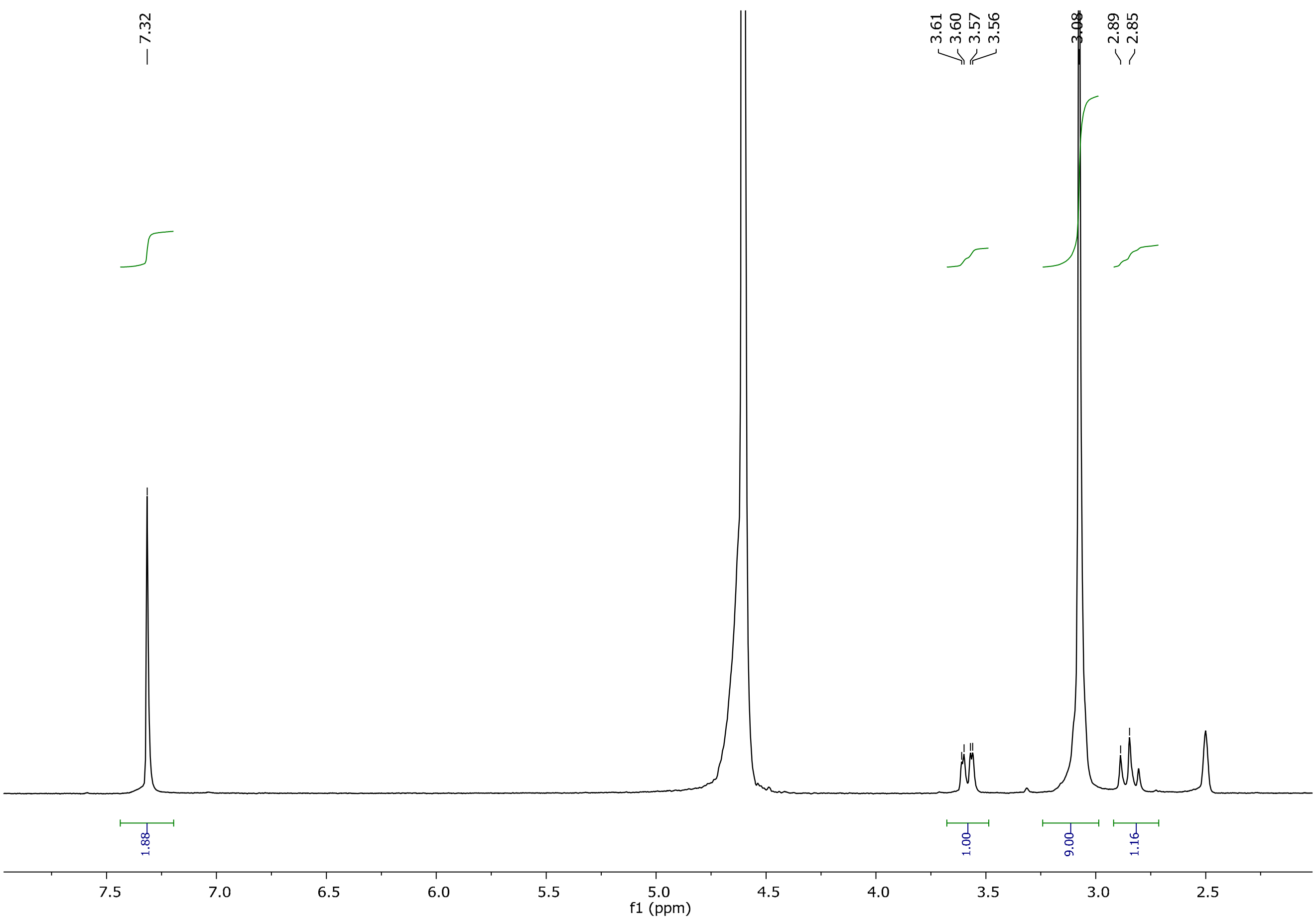

Supplementary figure S2. ^{1}H-RMN of TDB-3M in DMSO-d_6

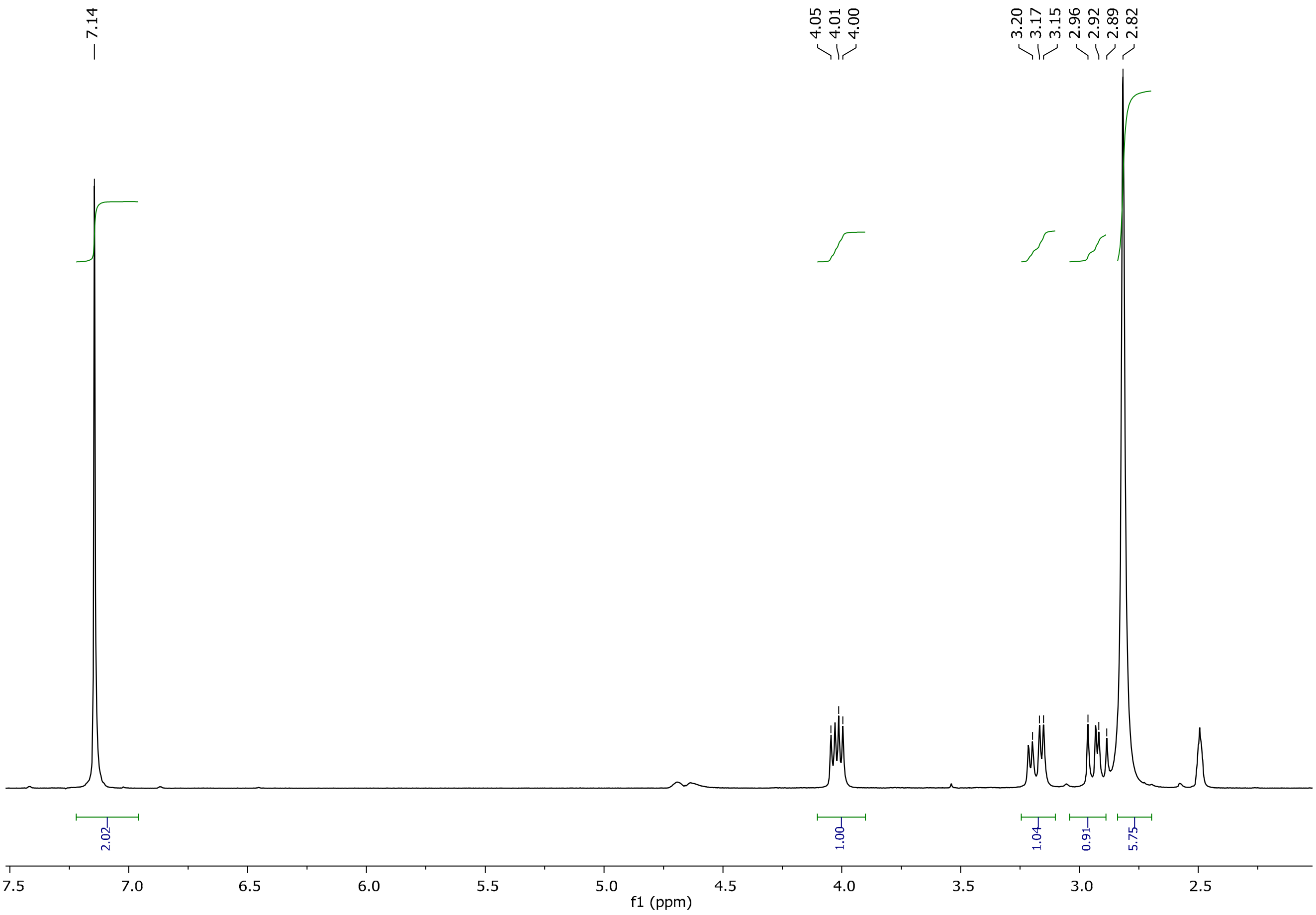

Supplementary figure S3. ^{1}H-RMN of TDC-2M in DMSO-d_6

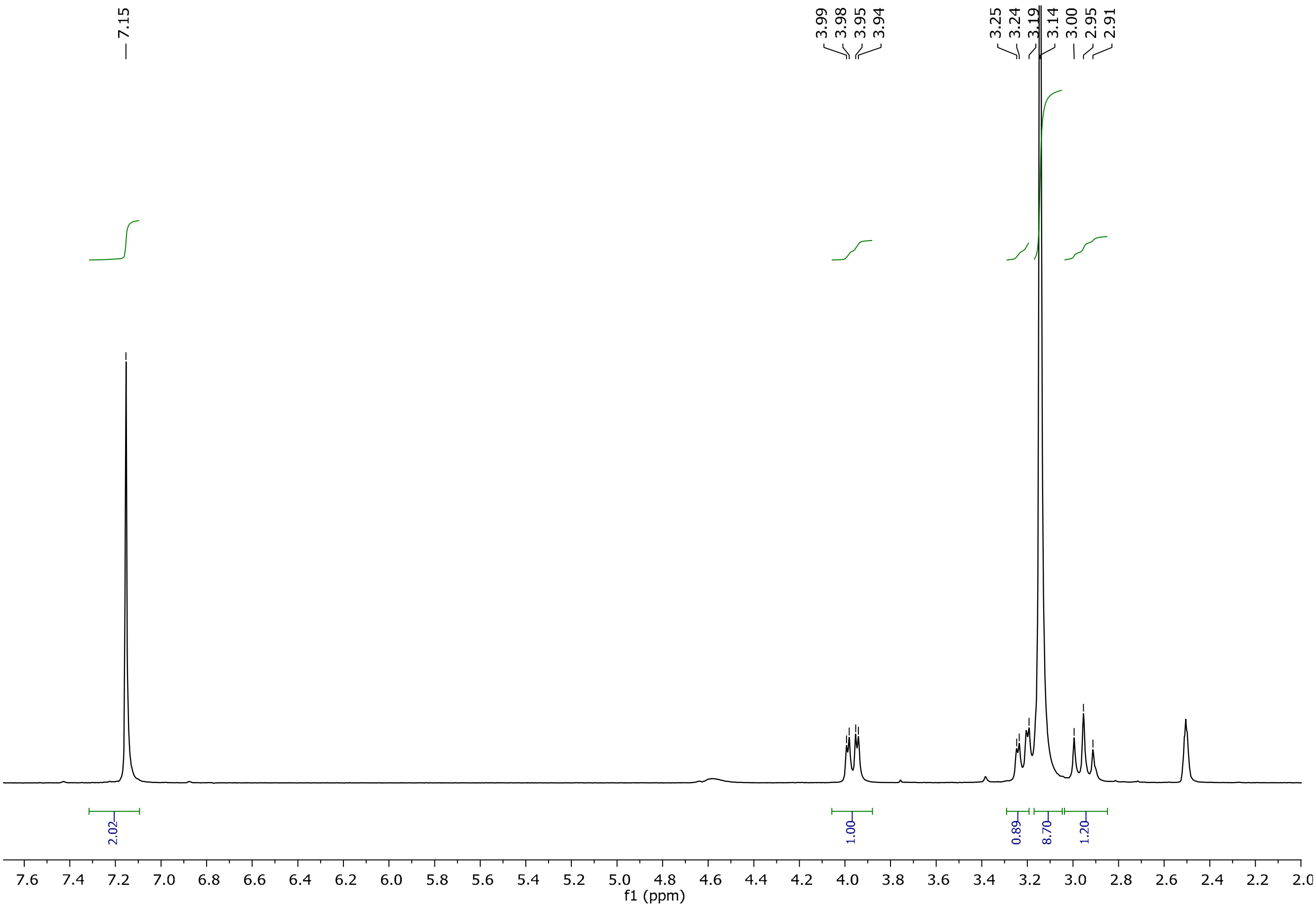

Supplementary figure S4. ^{1}H-RMN of TDC-3M in DMSO-d_6

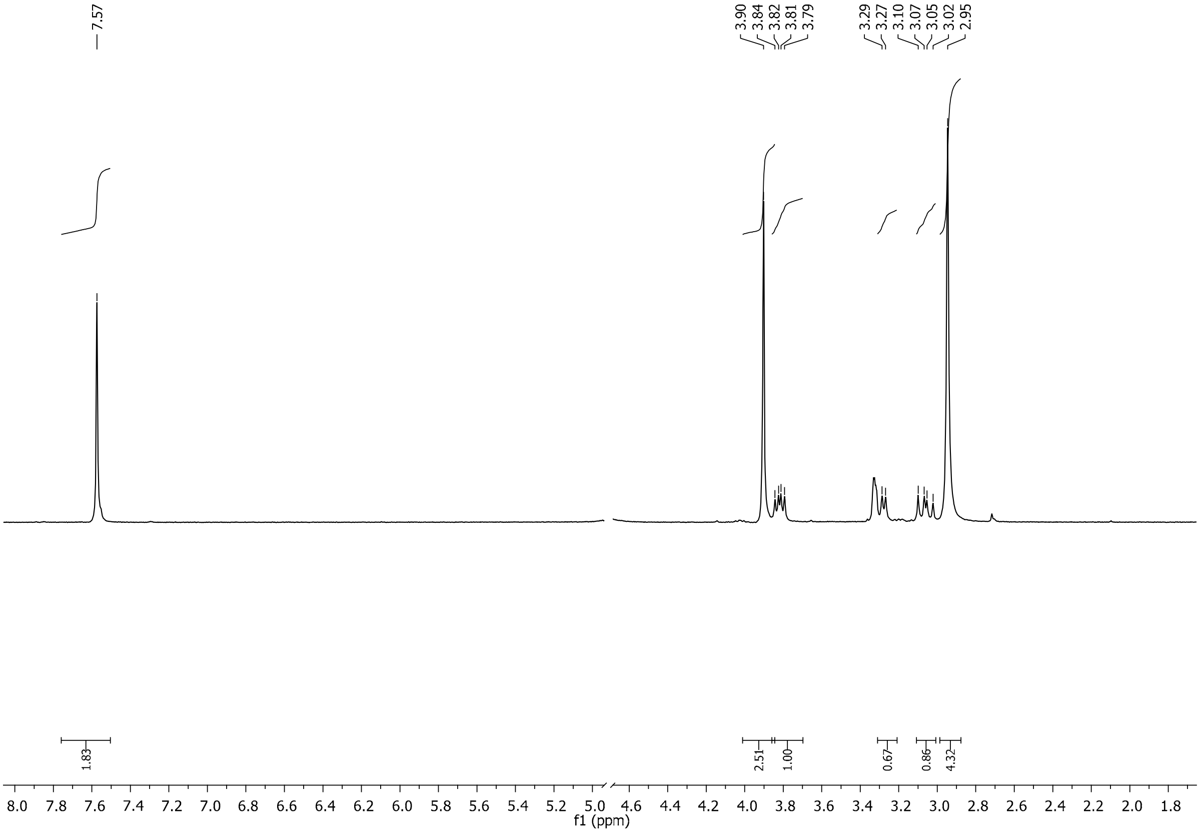

Supplementary figure S5. ^{1}H-RMN of TODB-2M. in DMSO-d_6

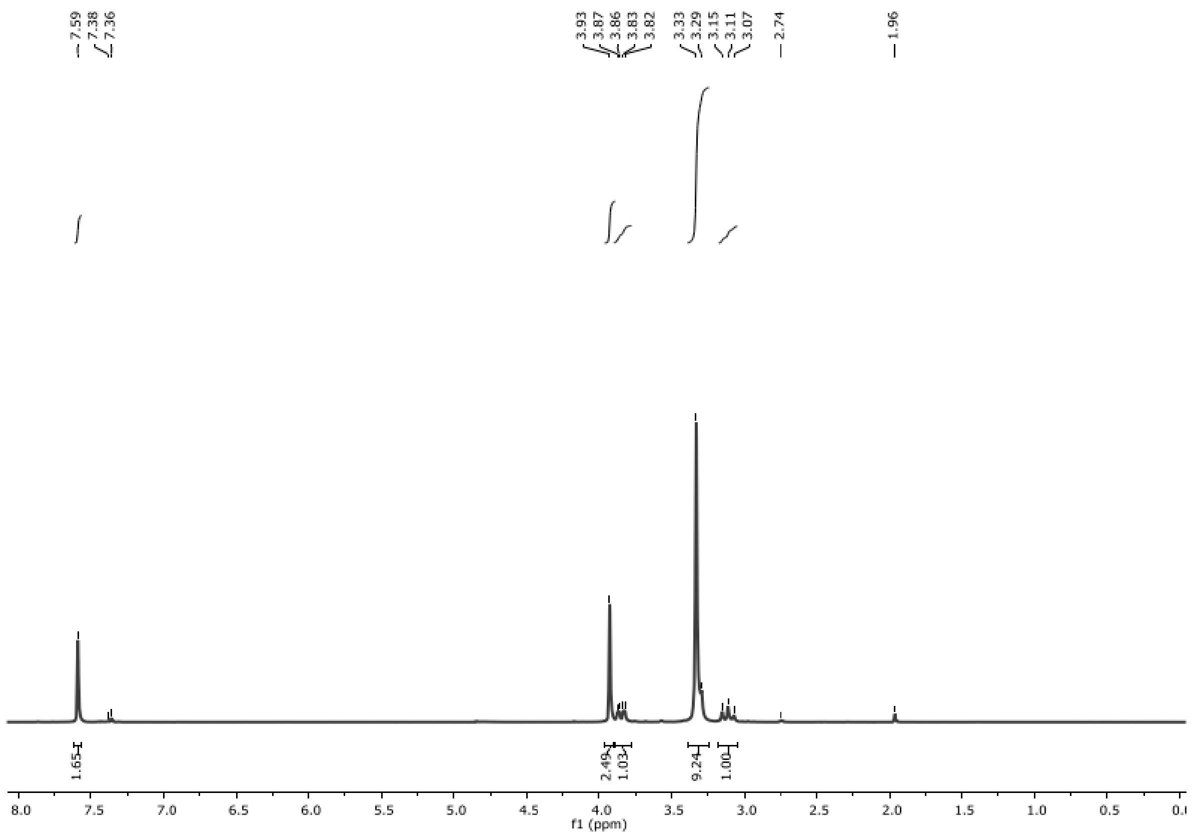

Supplementary figure S6. ^{1}H-RMN of TODB-3M in DMSO-d_6

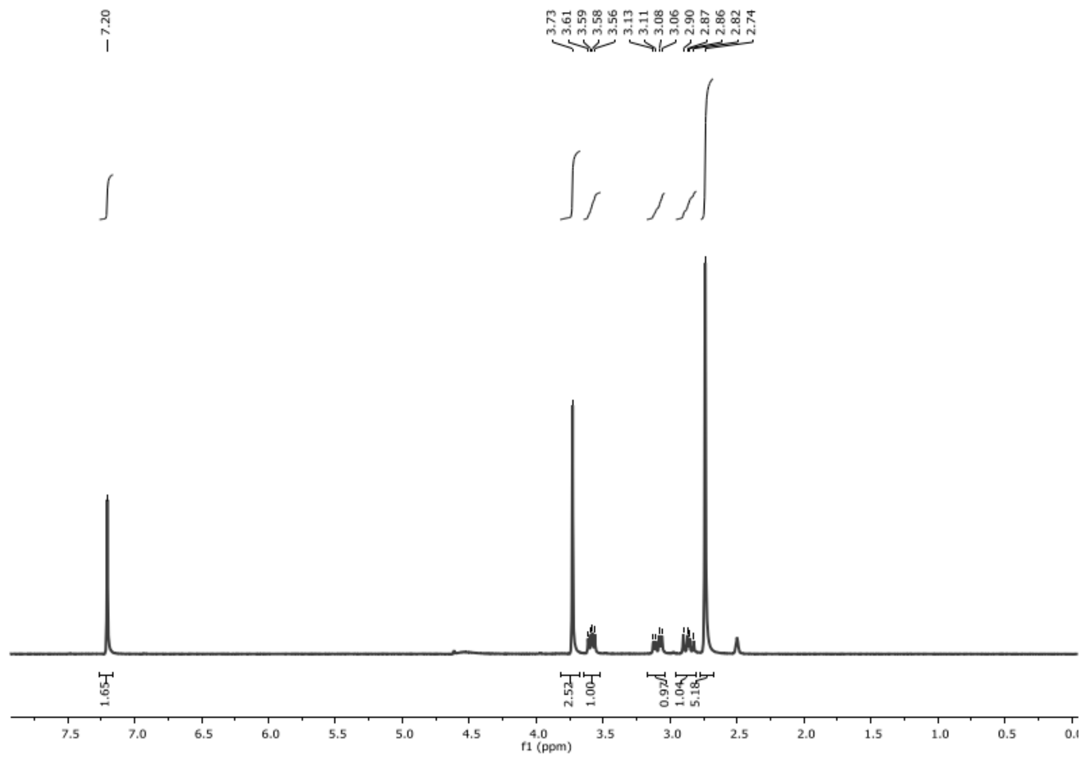

Supplementary figure S7. ^{1}H-RMN of TODC-2M in DMSO-d_6

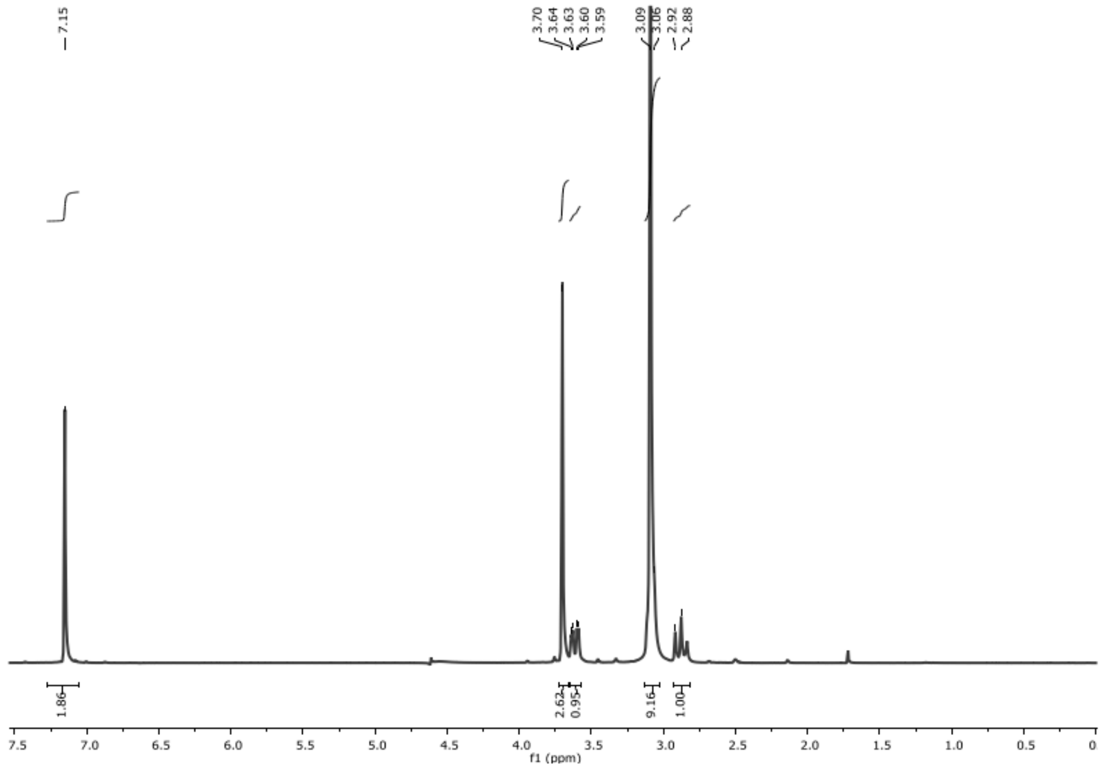

Supplementary figure S8. ^{1}H-RMN of TODC-3M. in DMSO-d_6

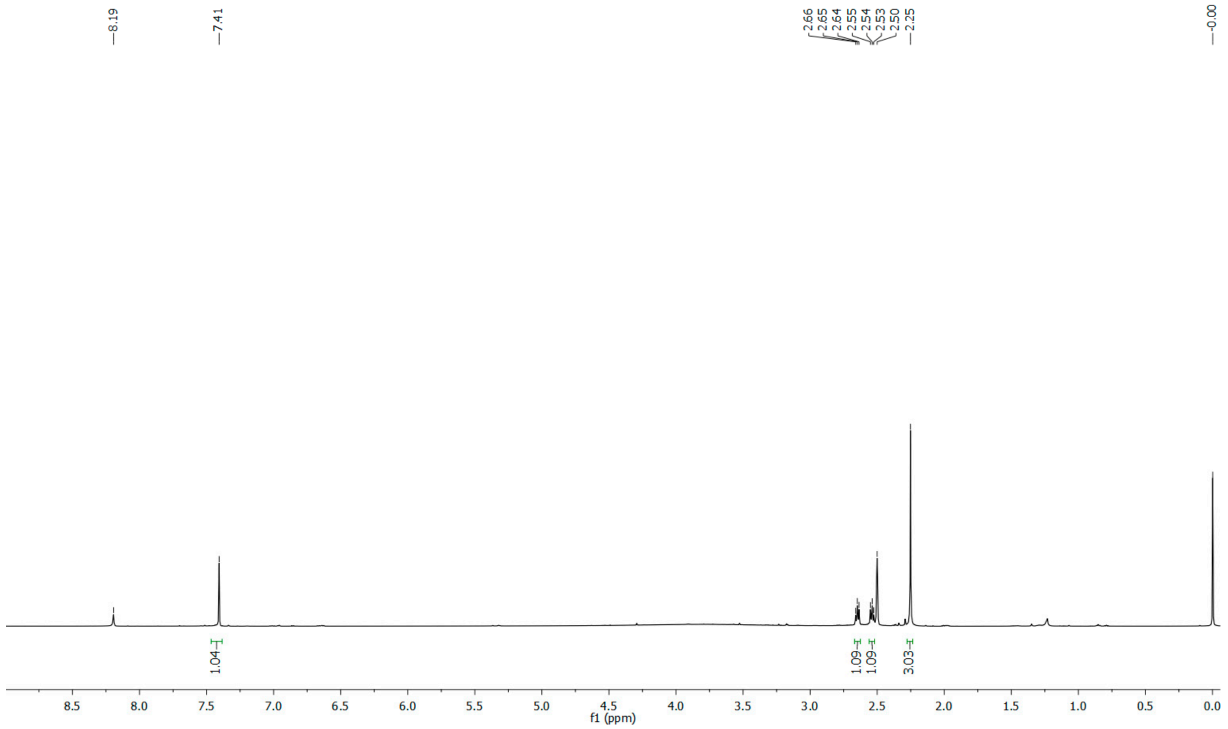

Supplementary figure S9. ^{1}H-RMN of YDB-2M in DMSO-d_6

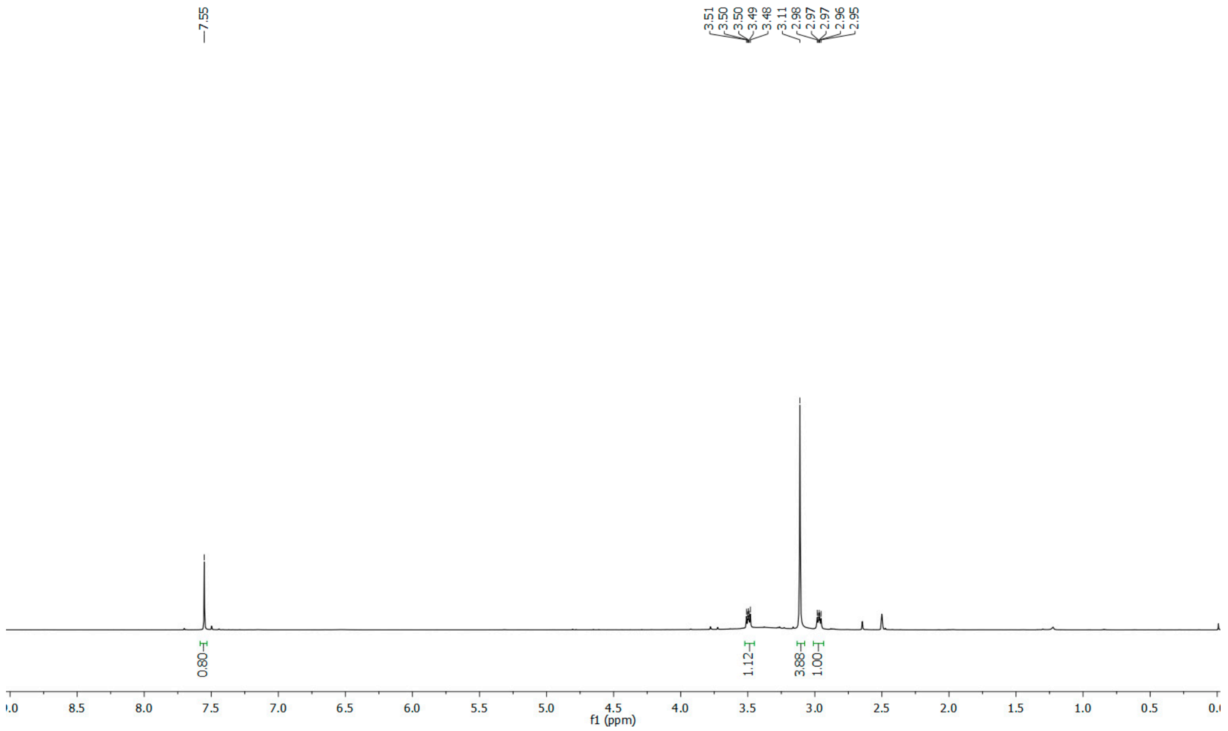

Supplementary figure S10. ^{1}H-RMN of YDB-3M in DMSO-d_6

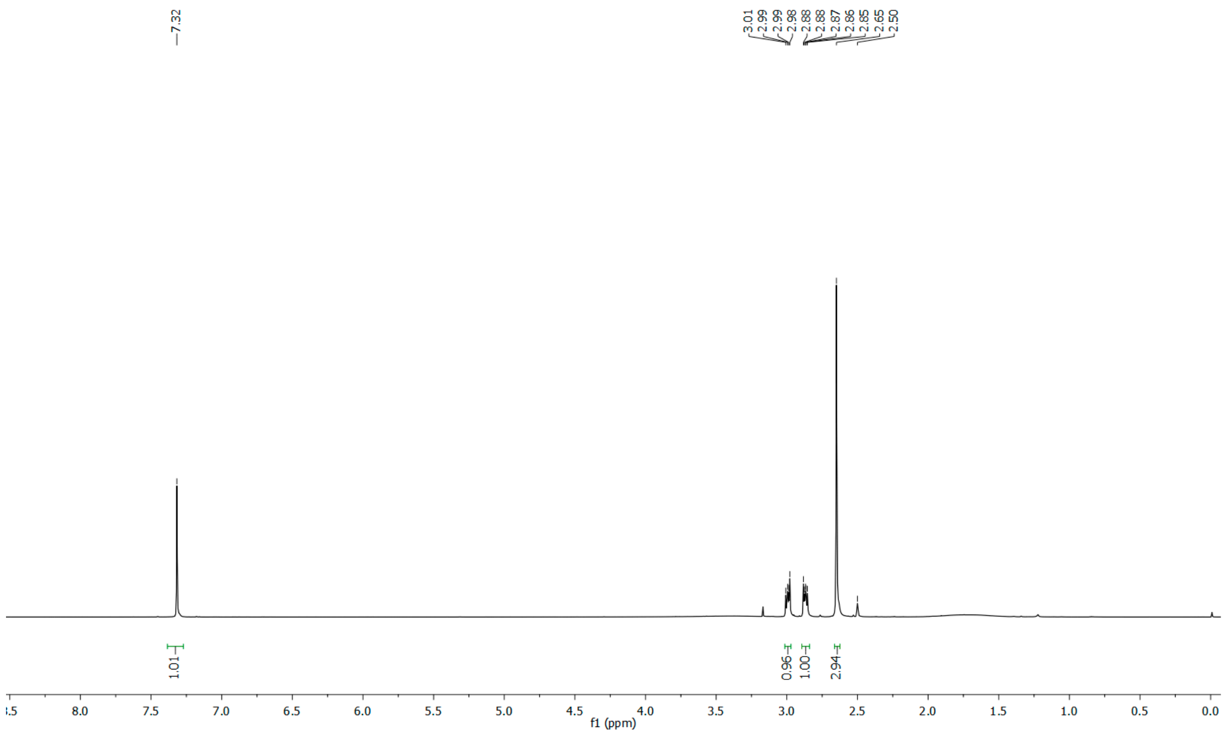

Supplementary figure S11. ^{1}H-RMN of YDC-2M in DMSO-d$_6$

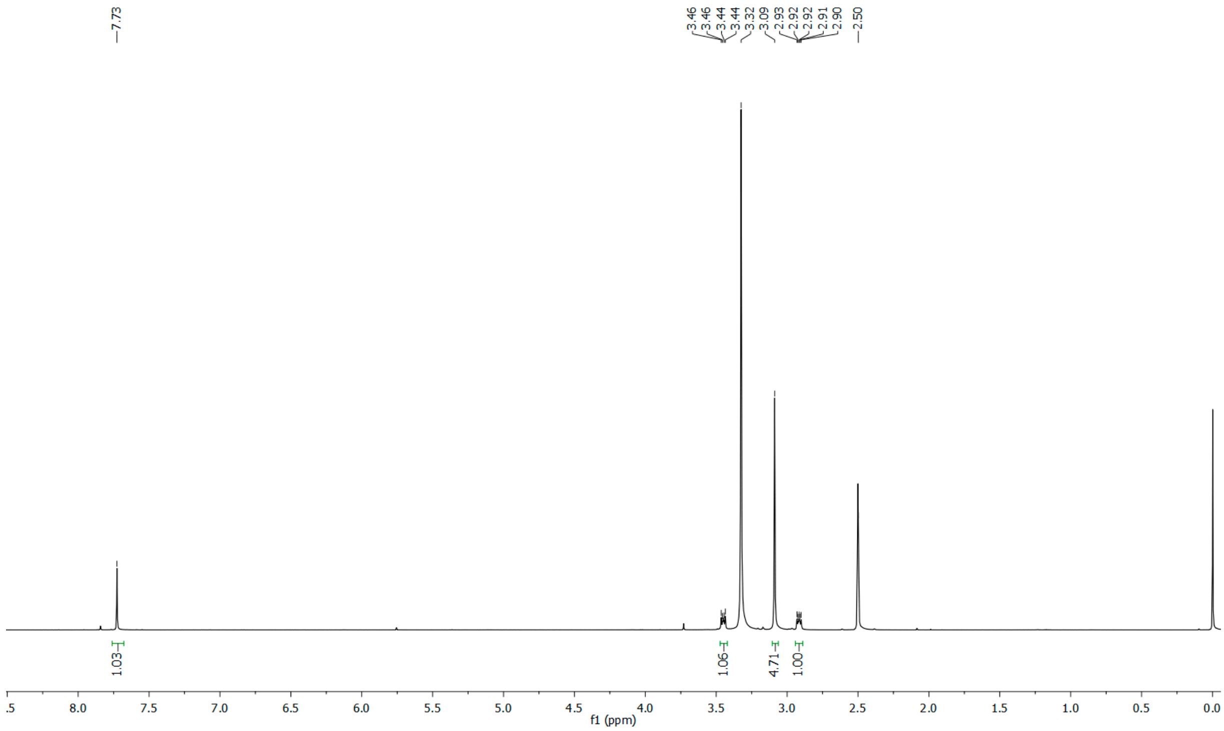

Supplementary figure S12. ^{1}H-RMN of YDC-3M in DMSO-d$_6$

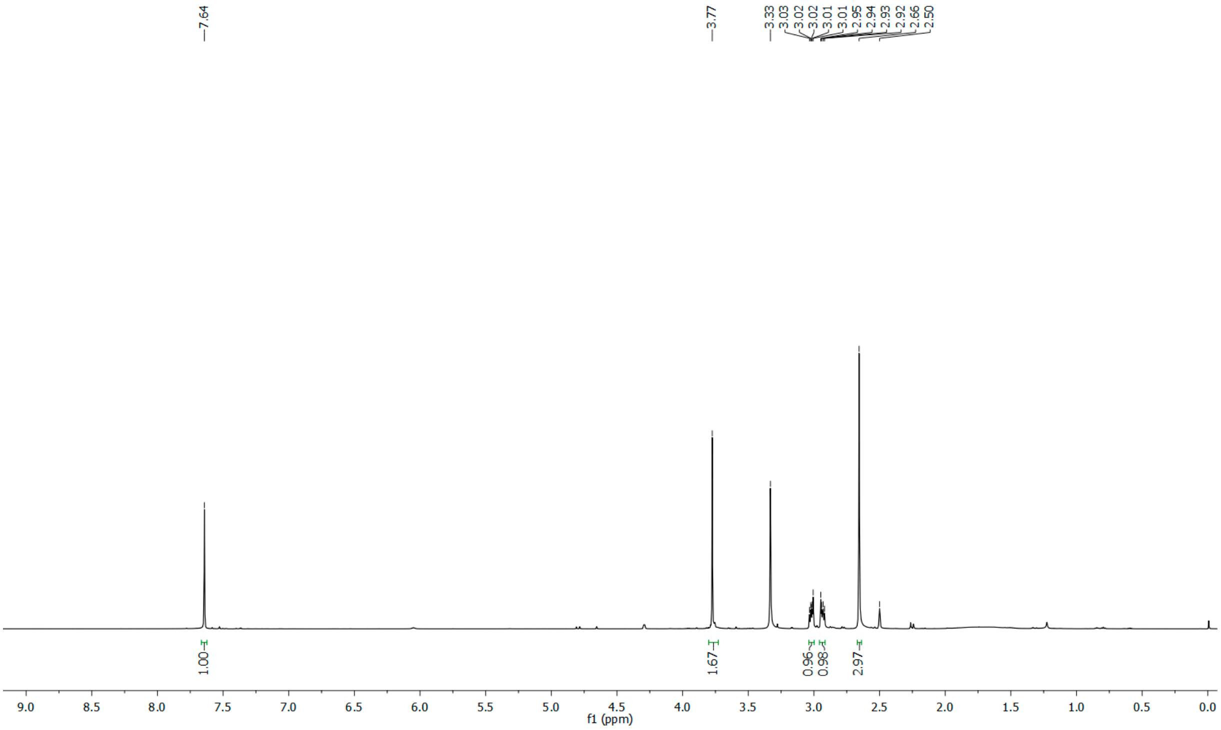

Supplementary figure S13. ¹H-RMN of YODB-2M in DMSO-d₆

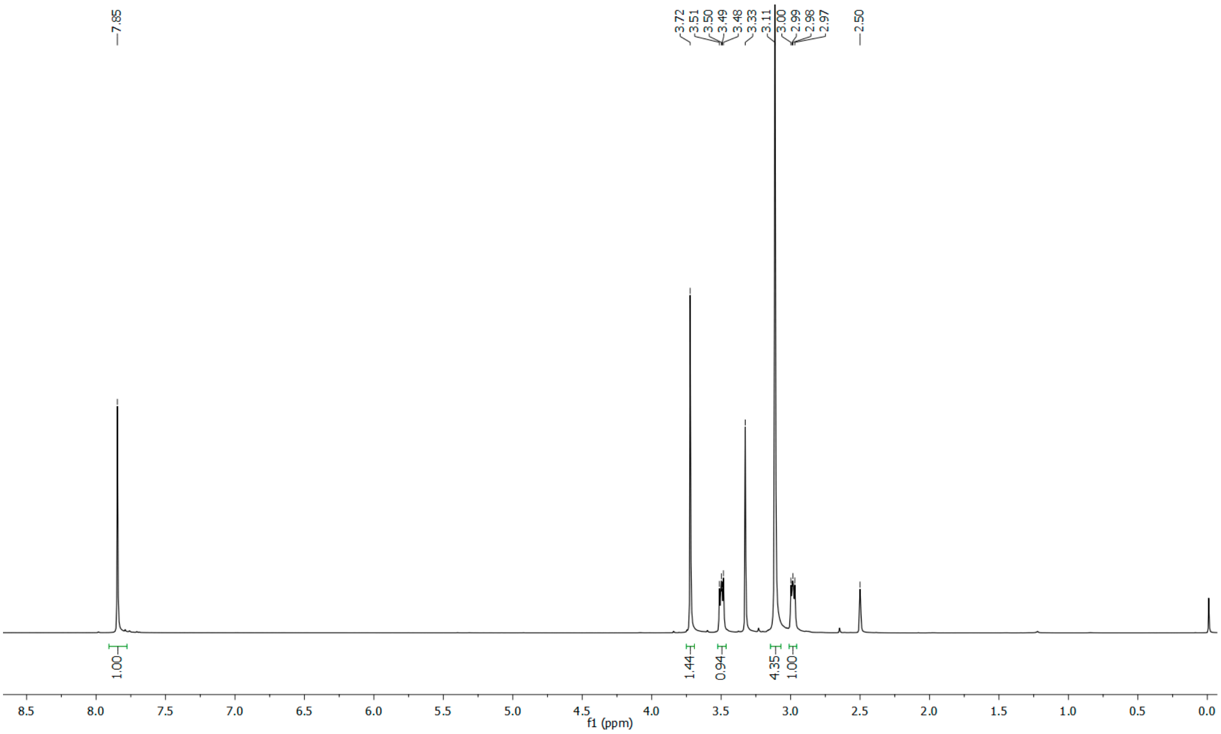

Supplementary figure S14. ¹H-RMN of YODB-3M in DMSO-d₆

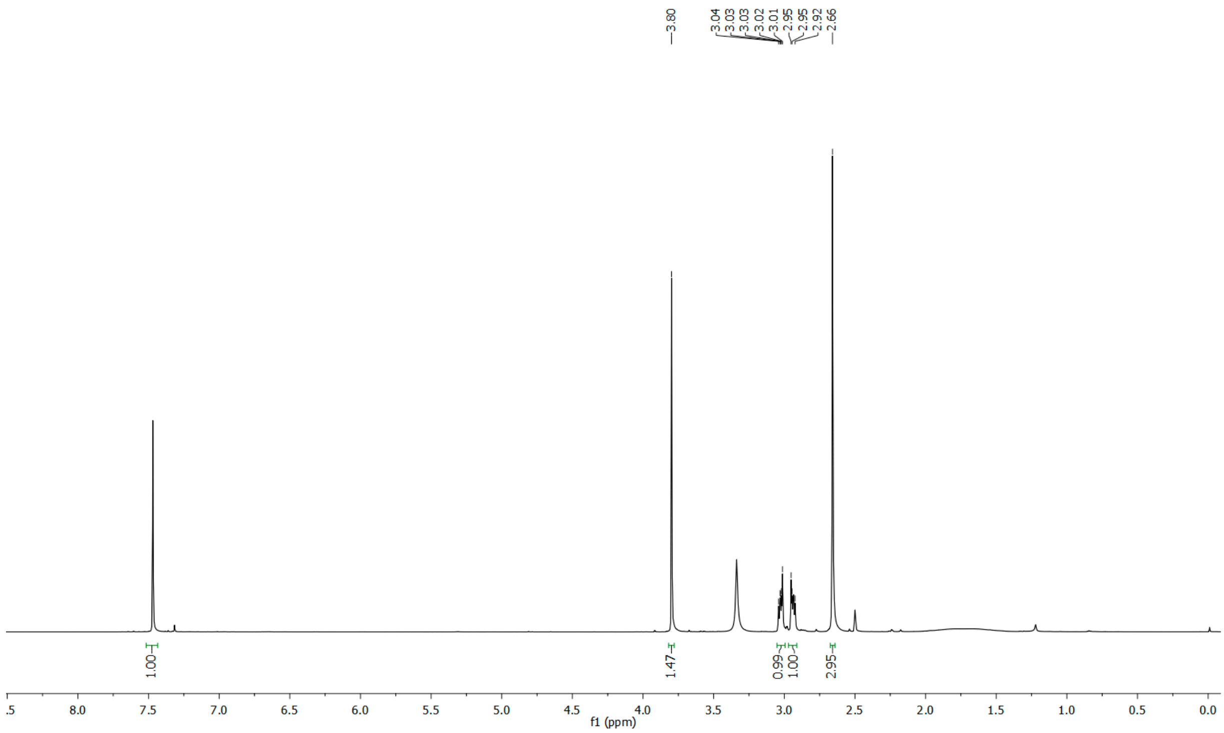

Supplementary figure S15. ^{1}H-RMN of YODC-2M in DMSO-d_6

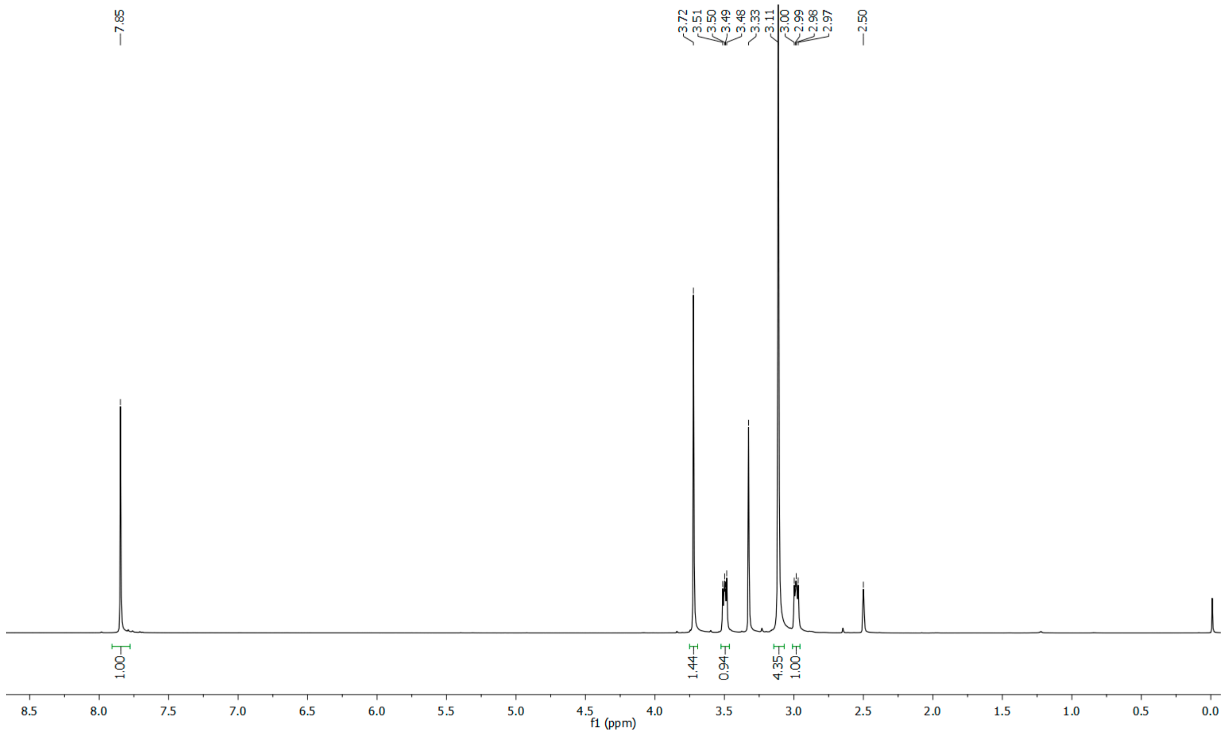

Supplementary figure S16. ^{1}H-RMN of YODC-3M in DMSO-d_6

	Mass	Hydrogen bond donor	Hydrogen bond acceptors	LogP	Molar Refractivity
**TDB-2M**	365	2	3	-0,28	68,82
**TDB-3M**	379	1	3	1,28	74,04
**TDC-2M**	278	2	3	-2,04	57,57
**TDC-3M**	292	1	3	-0,48	62,80
**TODB-2M**	379	1	3	0,03	73,70
**TODB-3M**	393	0	3	1,59	78,93
**TODC-2M**	292	1	3	-1,74	62,46
**TODC-3M**	306	0	3	-0,17	67,68
**YDB-2M**	322	2	1	1,60	64,89
**YDB-3M**	336	1	1	3,17	70,11
**YDC-2M**	235	2	1	-0,16	53,65
**YDC-3M**	249	1	1	1,40	58,87
**YODB-2M**	336	1	1	1,91	69,78
**YODB-3M**	350	0	1	3,47	75,00
**YODC-2M**	249	1	1	0,15	58,53
**YODC-3M**	263	0	1	1,71	63,76

Supplementary table S1. Di-halogenated compounds evaluated in the study satisfied Lipinski's rule of five. All compounds have a molecular mass of less than 500 Daltons, less than 5 hydrogen bond donors, less than 10 hydrogen bond acceptors, have high lipophilicity (expressed as LogP less than 5) and the molar refractivity was between 40-130.

Concentrations (μM)	300	150	75	37	18
**TDB-2M**	N/A	-51,9	-19,2	-45,5	-57,9
**TDB-3M**	N/A	-61,9	-18,0	-9,6	-20,5
**TDC-2M**	47,3*	32,3	37,9	33,0	N/A
**TDC-3M**	25,9	29,6*	39,7*	20,4	N/A
**TODB-2M**	N/A	45,8*	37,7	34,1	36,3
**TODB-3M**	N/A	-9,2	-17,7	-43,3	-26,0
**TODC-2M**	N/A	61,1*	60,5*	46,4*	38,0*
**TODC-3M**	59,9*	46,7*	37,6*	54,9*	N/A
**YDB-2M**	N/A	-45,8	7,0	2,7	26,1
**YDB-3M**	N/A	N/A	-845,8	-10,2	7,2
**YDC-2M**	N/A	-2,8	19,1	8,8	-4,1
**YDC-3M**	N/A	27,5	33,4	42,3*	50,2*
**YODB-2M**	N/A	-14,9	-88,5	-73,9	-51,1
**YODB-3M**	N/A	9,3	-9,0	-14,1	-25,1
**YODC-2M**	N/A	-96,2	26,9	12,2	24,5
**YODC-3M**	N/A	-1,3	-1,0	-7,7	-19,7

Supplementary table S2. Percentage inhibition of HIV-1 BaL replication of each di-halogenated compound. Each sample was tested in triplicate under two independent experiments. Compounds marked with * showed p-values less than 0.0001 when compared to the positive infection control and those marked with N/A have no statistical analysis available.

**Concentrations (μM)**	**150**	**75**	**37**	**18**
**TDB-2M**	2,9	-2,9	15,9	-1,3
**TDB-3M**	-22,4	-15,7	21,6	2,7
**TDC-2M**	-72,2	-39,5	-15,4	-4,8
**TDC-3M**	15,1	8,1	9,6	-110,9
**TODB-2M**	95,9*	9,9	-4,0	22,2
**TODB-3M**	-34,0	3,2	-41,1	12,0
**TODC-2M**	99,6*	62,8	40,6	-18,5
**TODC-3M**	14,9	26,7	-6,9	-1,3
**YDB-2M**	-20,7	9,8	-7,0	14,6
**YDB-3M**	N/A	-19,4	1,8	39,2*
**YDC-2M**	29,1	22,6	-22,0	15,1
**YDC-3M**	29,9	31,2	-25,7	41,0*
**YODB-2M**	-17,0	-20,2	-20,0	-24,6
**YODB-3M**	13,1	20,3	-10,5	-3,1
**YODC-2M**	2,5	-2,2	2,3	-0,3
**YODC-3M**	0,5	-8,6	-5,8	3,2

Supplementary table S3. Percentage inhibition of HIV-1IIIB (X4 strain) replication of each di-halogenated compound. Each sample was tested in triplicate under two independent experiments. Compounds marked with * showed p-values less than 0.001 when compared to the positive infection control and those marked with N/A have no statistical analysis available.

	PR	RT	gp120	p24	p17	IN	gp41
**TDB-2M**	-9,10 ± 0,00	-6,90 ± 0,00	-4,87 ± 0,19	-5,20 ± 0,00	-5,33 ± 0,09	-4,87 ± 0,38	-3,90 ± 0,00
**TDB-3M**	-9,20 ± 0,00	-6,23 ± 0,09	-4,50 ± 0,14	-5,20 ± 0,00	-5,10 ± 0,00	-4,43 ± 0,05	-3,90 ± 0,00
**TDC-2M**	-9,00 ± 0,00	-7,20 ± 0,00	-5,17 ± 0,66	-5,50 ± 0,08	-5,37 ± 0,05	-5,03 ± 0,38	-4,00 ± 0,00
**TDC-3M**	-9,30 ± 0,00	-6,67 ± 0,05	-4,90 ± 0,00	-5,40 ± 0,00	-5,30 ± 0,00	-4,60 ± 0,00	-4,00 ± 0,00
**TODB-2M**	-9,07 ± 0,05	-6,40 ± 0,00	-4,77 ± 0,24	-5,30 ± 0,00	-5,33 ± 0,09	-4,47 ± 0,05	-3,83 ± 0,09
**TODB-3M**	-9,30 ± 0,00	-6,13 ± 0,05	-4,23 ± 0,09	-5,40 ± 0,00	-5,07 ± 0,05	-4,53 ± 0,05	-3,90 ± 0,00
**TODC-2M**	-9,30 ± 0,00	-6,73 ± 0,09	-4,67 ± 0,05	-5,50 ± 0,08	-5,03 ± 0,05	-4,77 ± 0,31	-4,00 ± 0,00
**TODC-3M**	-9,37 ± 0,09	-6,10 ± 0,00	-4,83 ± 0,24	-5,60 ± 0,00	-5,07 ± 0,05	-4,50 ± 0,00	-3,90 ± 0,00
**YDB-2M**	-8,17 ± 0,05	-6,53 ± 0,05	-5,03 ± 0,17	-5,20 ± 0,00	-5,00 ± 0,00	-4,63 ± 0,05	-3,80 ± 0,00
**YDB-3M**	-8,50 ± 0,00	-6,53 ± 0,05	-4,93 ± 0,45	-5,10 ± 0,00	-4,73 ± 0,05	-4,20 ± 0,08	-3,80 ± 0,00
**YDC-2M**	-8,30 ± 0,00	-6,97 ± 0,05	-6,10 ± 0,00	-5,20 ± 0,00	-4,80 ± 0,00	-4,67 ± 0,12	-3,97 ± 0,05
**YDC-3M**	-8,50 ± 0,00	-6,50 ± 0,00	-5,57 ± 0,76	-5,07 ± 0,05	-4,70 ± 0,00	-4,40 ± 0,14	-3,83 ± 0,05
**YODB-2M**	-8,20 ± 0,00	-6,53 ± 0,05	-4,73 ± 0,17	-5,00 ± 0,00	-4,90 ± 0,00	-4,63 ± 0,09	-3,73 ± 0,05
**YODB-3M**	-8,40 ± 0,00	-6,20 ± 0,00	-5,17 ± 0,05	-5,10 ± 0,00	-4,70 ± 0,00	-4,30 ± 0,08	-3,53 ± 0,05
**YODC-2M**	-8,40 ± 0,00	-6,80 ± 0,00	-6,23 ± 0,05	-5,20 ± 0,00	-4,73 ± 0,05	-4,60 ± 0,14	-3,93 ± 0,05
**YODC-3M**	-8,53 ± 0,05	-6,20 ± 0,00	-5,57 ± 0,90	-5,20 ± 0,00	-4,80 ± 0,00	-4,33 ± 0,05	-3,60 ± 0,00
**Fostemsavir**			-6,23 ± 0,40				
**Abacavir**		-7,03 ± 0,09					
**Zidovudine**		-7,87 ± 0,05					
**Efavirenz**		-10,1 ± 0,00					
**Lopinavir**	-14,97 ± 0,0						
**Ritonavir**	-13,73 ± 0,0						
**Dolutegravir**						-7,50 ± 0,08	
**Raltegravir**						-7,33 ± 0,24	

Supplementary table S4. Binding energies between compounds and viral proteins and study control drugs. Free binding energies were obtained by molecular docking with AutodockVina®. Each interaction was analyzed in triplicate; data represent the mean ± standard deviation. gp120 (glycoprotein 120), RT (Reverse transcriptase), gp41 (glycoprotein 41), PR (protease), IN (integrase), p17 (matrix protein), p24 (capsid protein).

Protein	Compound	Interactions	
		Polar	Non-polar
RT	TODB-2M	Tyr318	Pro95, Leu100, Lys101, Lys103, Val106, Val179, Tyr181, Phe227, Trp229, Leu234, His235, Pro236
	TODC-2M	Tyr181, Tyr188, Trp229, Tyr318	Leu100, Lys101, Lys102, Lys103, Val106, Tyr183, Phe227, Leu234, His235, Pro236
	TODC-3M	Lys101	Pro95, Leu100, Lys103, Val106, Val179, Ile180, Tyr181, Tyr188, Gly190, Phe227, Trp229, Leu234
	YDC-3M		Pro95, Leu100, Lys101, Lys103, Val106, Tyr181, Tyr188, Phe227, Trp229, Leu234, His235, Pro236, Tyr318
PR	TODB-2M	Asp25, Asp25', Asp29', Ile50	Arg8, Gly27, Gly27', Ala28, Ala28', Asp30', Val32', Ile47', Gly48, Gly49, Gly49', Ile50', Pro81', Ile84, Ile84'
	TODC-2M		Arg8, Gly27, Gly27', Ala28, Ala28', Asp30', Val32', Ile47', Gly48', Gly49, Gly49', Ile50', Pro81', Ile84, Ile84'
	TODC-3M		Arg8, Leu23', Gly27, Gly27', Ala28, Ala28', Asp30', Val32', Ile47', Gly48, Gly48', Gly49, Gly49', Ile50', Ile84, Ile84'
	YDC-3M	Asp25, Asp25', Asp30	Gly27, Ala28, Asp29, Val32, Ile47, Gly49, Gly49', Ile50, Ile50', Ile76, Ile84, Ile84'
Gp120	TODC-3M		Trp112, Val255, Ser256, Thr257, Ile371, Ser375, Phe376, Phe382, Ile424, Asn425, Met426, Trp427, Gly473, Met475

Supplementary table S5. Interactions of the promising compounds with RT/PR/gp120. The table describes the polar (hydrogen bonds, salt bridges and pi-anionic interactions) and non-polar (van der Waals, alkyl, pi-alkyl and Pi-sigma) interactions between the amino acids of the different proteins and the different ligands.

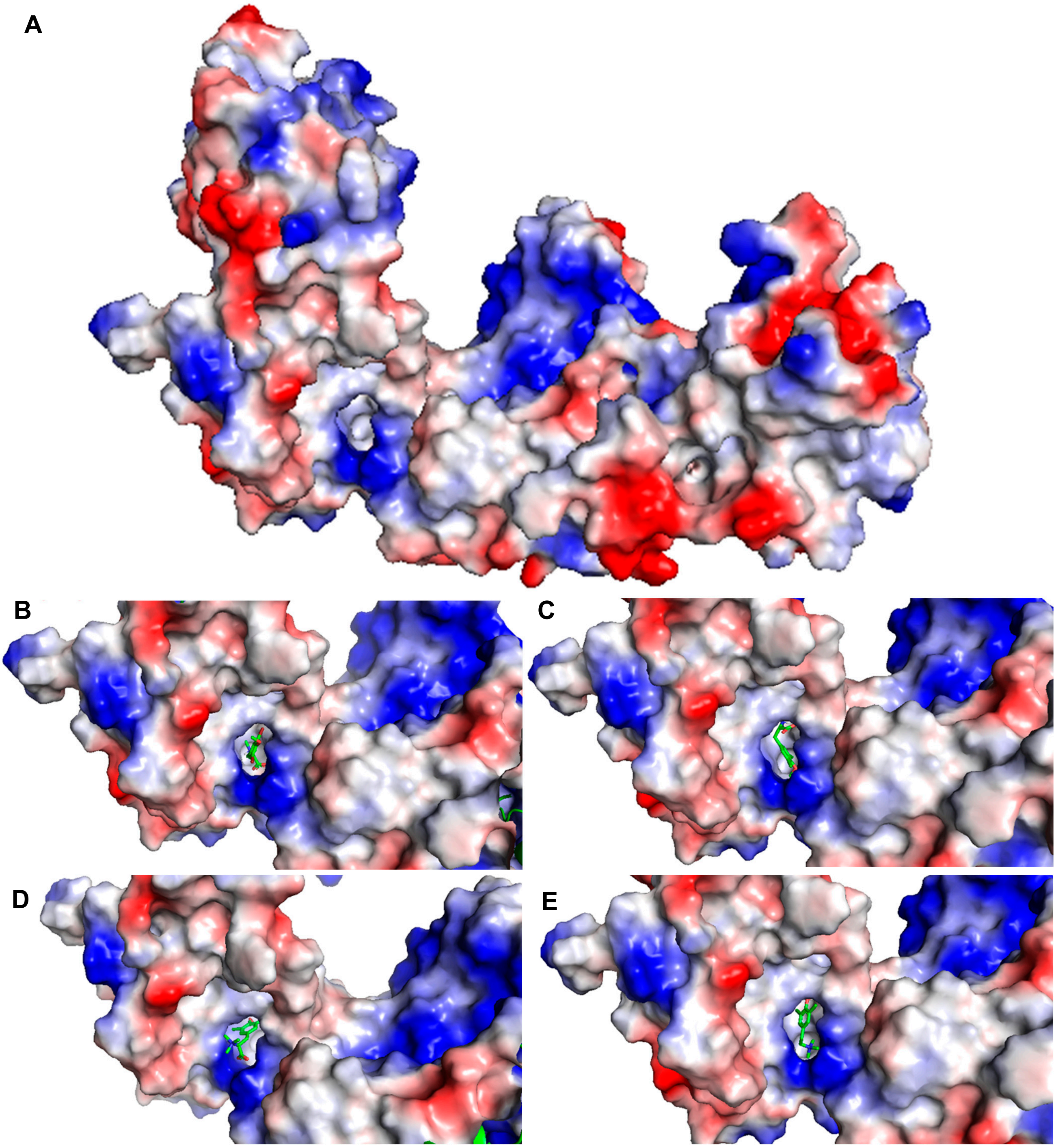

Supplementary figure S17. Binding of di-halogenated compounds to the active site region of reverse transcriptase. The interaction site of RT with (B) TODB-2M, (C) TODC-2M, (D) TODC-3M and (E) is shown. The red areas of the surface represent the acid regions, the white areas represent the neutral, and the blue areas are the basic regions. Images were obtained using PyMOL.

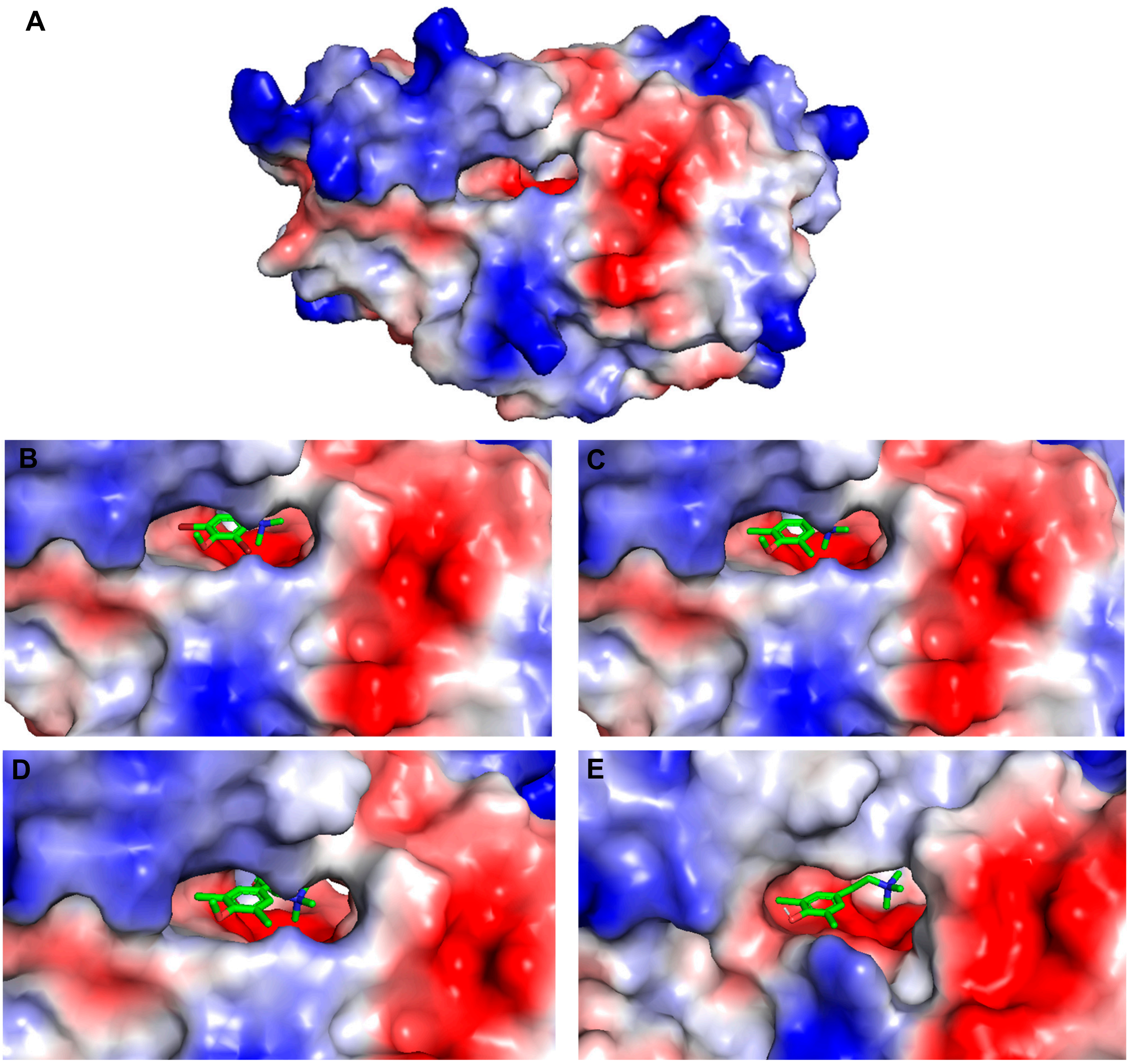

Supplementary figure S18. Binding of di-halogenated compounds to the active site region of protease. The interaction site of PR with (B) TODB-2M, (C) TODC-2M, (D) TODC-3M and (E) is shown. The red areas of the surface represent the acid regions, the white areas represent the neutral, and the blue areas are the basic regions. Images were obtained using PyMOL.

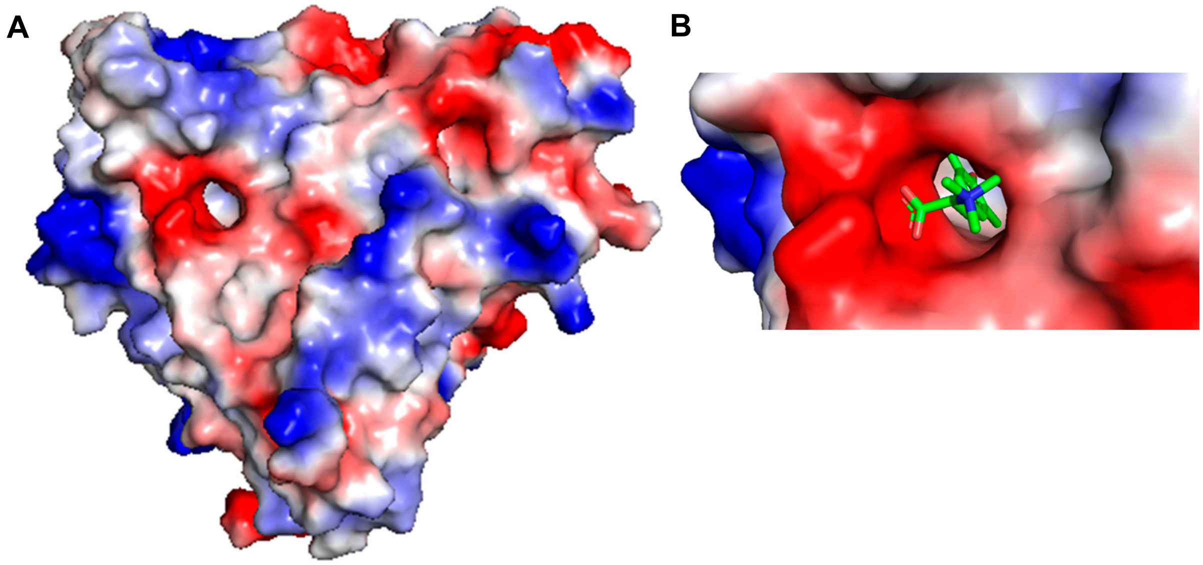

Supplementary figure S19. Binding of di-halogenated compounds to the active site region of gp120. The interaction site of gp120 with (B) TODC-3M is shown. The red areas of the surface represent the acid regions, the white areas represent the neutral, and the blue areas are the basic regions. Images were obtained using PyMOL.
